# Recent Progress in Conducting Polymers for Hydrogen Storage and Fuel Cell Applications

**DOI:** 10.3390/polym12112480

**Published:** 2020-10-26

**Authors:** Neelima Mahato, Hyeji Jang, Archana Dhyani, Sunghun Cho

**Affiliations:** 1 School of Chemical Engineering, Yeungnam University, Gyeongsan 38541, Korea; neelapchem@gmail.com (N.M.); sdfg8396@gmail.com (H.J.); 2 Department of Applied Sciences, School of Engineering, University of Petroleum and Energy Studies, Dehradun, Uttarakhand 248007, India; adhyani@ddn.upes.ac.in

**Keywords:** conducting polymers, polyaniline, polypyrrole, polythiophene, hydrogen storage, fuel cell, proton conducting solid polymer electrolytes

## Abstract

Hydrogen is a clean fuel and an abundant renewable energy resource. In recent years, huge scientific attention has been invested to invent suitable materials for its safe storage. Conducting polymers has been extensively investigated as a potential hydrogen storage and fuel cell membrane due to the low cost, ease of synthesis and processability to achieve the desired morphological and microstructural architecture, ease of doping and composite formation, chemical stability and functional properties. The review presents the recent progress in the direction of material selection, modification to achieve appropriate morphology and adsorbent properties, chemical and thermal stabilities. Polyaniline is the most explored material for hydrogen storage. Polypyrrole and polythiophene has also been explored to some extent. Activated carbons derived from conducting polymers have shown the highest specific surface area and significant storage. This review also covers recent advances in the field of proton conducting solid polymer electrolyte membranes in fuel cells application. This review focuses on the basic structure, synthesis and working mechanisms of the polymer materials and critically discusses their relative merits.

## 1. Introduction

### 1.1. Hydrogen Storage

Hydrogen is the most abundant element in the universe, comprising ~75% of the chemical composition. It is considered among the most efficient sources of renewable energy in current times as the excessive usage of fossil fuels has created huge problems for both the planet and life on the Earth, in terms of pollution and climate change owing to excessive mining, deforestation, extinction of wild life species (both plants and animals), oil spillage in the oceans and coastal lands, bleaching and dying of corals (coral reefs), rise in ocean levels and submerge of small islands. This has not only altered the very fundamental structure of the ecosystems but also pushed life on the earth towards an inevitable plunge of disharmony and innumerable diseases. The rise in the demands for a potential source of renewable energy in this scenario is undeniable and in grave need. Hydrogen possesses a substantially larger energy density per unit mass of 142 MJ/kg compared to other fossil fuels or hydrocarbon fuel [1]. Hence, hydrogen can offer a promising candidature as a pollution-free resource of renewable energy in the future times to come. The main challenges in the direction of domestication of hydrogen as a practical fuel alternative is its storage which must be reversible on board as and when required. The storage capacity of a particular material for hydrogen gas is defined as reversible gravimetric (wt.%) and volumetric (kg/m^3^) capacities. To make the hydrogen fuel cell powered vehicles (FCV) commercially operational, the challenges associated with its storage has to be addressed adequately. To achieve a low volumetric energy density for a workable fuel for vehicles, it requires additional safety measures, i.e., the vessel must be capable of withstanding high pressures of ~70–100 MPa. Furthermore, the entire design must be cost effective and fuel efficient in terms of mileage. FCVs can drive up to 400 km distance using 4 kg of hydrogen as any conventional vehicle running on gasoline can do using 24 kg of petroleum. In this direction, the gravimetric and volumetric hydrogen storage system capacities are investigated in view of the technical requirement targets. The gravimetric and volumetric hydrogen storage system capacity targets was first launched by USDOE (The United States Department of Energy) and USCAR (The United States Council for Automotive Research) in the year 2003 and revised in the year 2009 [2]. The DOE target for the year 2020 in terms of system gravimetric capacity is 0.045 kg(H_2_)/kg (system) and the system volumetric capacity is 0.030 kg(H_2_)/L-system. Moreover, the targets for the year 2025 are 0.055 kg(H_2_)/kg (System) and 0.040 kg(H_2_)/L-system for system gravimetric and system volumetric capacities, respectively [3].

### 1.2. Hydrogen Fuel Cells

Fuel cells are future power devices. These convert chemical energy stored in fuel (hydrogen or methane) to electrical energy. Fuel cells have an edge over combustion engines, in terms of producing electricity The by-products of fuel cells are water and heat which are pollution free. Furthermore, the heat energy produced can further be utilized in domestic applications. Fuel cells find a variety of applications, ranging from utility power stations to charging alaptop or a mobile cell phone, which require less power. At present, fuel cells are in the developing stage and huge focuses as well as funds are invested to gain and share maximum possible fundamental knowledge among the hard-working researchers to develop low-cost fuel cells that can deliver high performance and trustworthy durability. The technical targets set by the DOE for proton conductivities of the membrane to be (a) > 0.1 S cm^−1^ (at 393 K); 0.07 Scm^−1^ (at 298 K); and 0.01 S cm^−1^ at 253 K, and (b) area-specific resistance to be less than 0.02 Ohm cm^2^. (c) For a large scale commercialization of hydrogen fuel cell, the membrane has to be flat with a thickness between 50 and 100 µm, dense with minimum porosity, and (d) durability in terms of chemical and mechanical stability up to 5000 h above 353 K [4].

**Figure 1:**
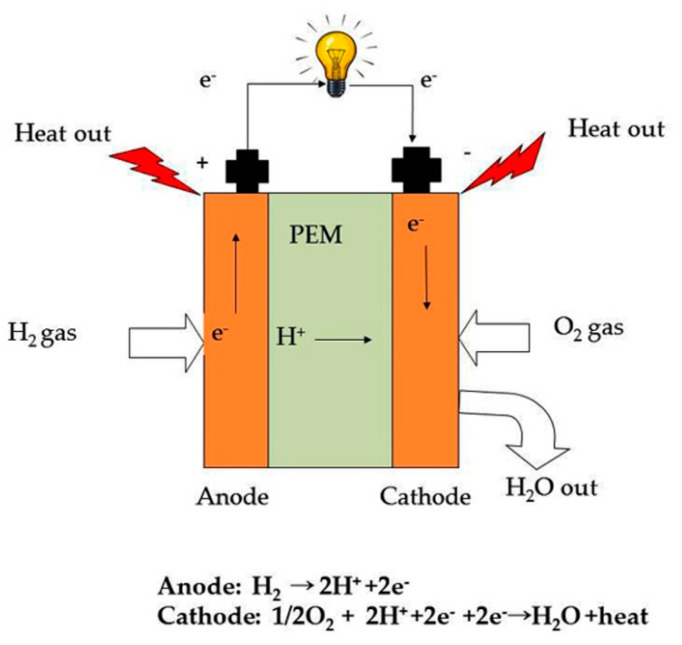
A typical hydrogen fuel cell consists of an anode, a cathode and a polymer electrolyte membrane (PEM) in the middle.

Based on the electrolyte membrane material, fuel cells have been categorized into (a) polymer electrolyte membrane fuel cells (PEMFCs), (b) alkaline fuel cells (AFCs), (c) solid oxide fuel cells (SOFCs), (d) molten carbonate fuel cells (MCFCs), and so on. A typical hydrogen fuel cell setup consists of two electrodes, an anode, a cathode, and a proton conducting membrane between them. The fuel cell is facilitated with hydrogen and oxygen supply to function and produce electricity. The hydrogen gas is fed to the anode and the catalyst (e.g., platinum containing compounds) present on the outer surface of the membrane strip off electrons from the hydrogen atoms at the electrode (anode). These electrons travel across an external circuit and generate electricity. The remaining protons pass through an electrolyte (which is a polymer membrane) towards the cathode. At cathode, the protons combine with oxygen and electrons to produce water and heat (Figure 1). The electrolyte in a hydrogen fuel cell is a solid membrane, also called a solid polymer electrolyte (SPE), which was first introduced by Gemini space program during the early 1960s [5]. Alternately, it is termed as polymer electrolyte membrane (PEM).

In hydrogen fuel cells, polymer electrolyte membranes (PEMs), particularly proton conducting polymer electrolytes, are popular due to their simple and compact design, enabling the cell to deliver high power densities [6]. There are two important functions of the electrolyte membranes: (a) it acts as a barrier which separates the hydrogen and oxygen from direct mixing, and (b) it transports or “conducts” protons from anode to cathode.

Therefore, the membrane material must be a “proton conducting” material. The important material properties of a solid polymer electrolyte to function satisfactorily as a polymer electrolyte membrane are: (a) high proton conductivity; (b) low electronic conductivity; (c) low permeability for fuel gases and the oxidant (i.e., it should not allow the crossover of hydrogen and oxygen); (d) adequate chemical, electrochemical, mechanical and thermal stabilities in a harsh fuel cell environment; (e) appreciable hydrolytic stability under operational conditions (which produces water and peroxides that might degrade the polymer material); (f) should allow adequate water transport (via diffusion and electro-osmotic); (g) Significant stability in terms of the dimensional aspects and morphology of the polymeric material; (h) long life time; and (i) inexpensive or low cost fabrication [7].

## 2. Materials for Hydrogen Storage

**Figure 2:**
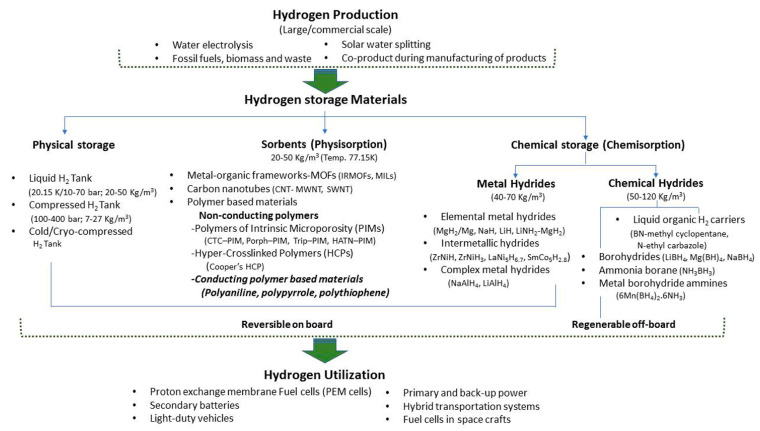
Hydrogen storage materials. (IRMOFS- Isoreticular metal–organic frameworks; MILs- ***M*** *atériaux de l’* ***I*** *nstitut* ***L*** *avoisier*; MWNT-Multi-walled carbon nanotubes; SWNT- Single-walled carbon nanotubes; PIM-Polymers of Intrinsic Microporosity; CTC–PIM-Cyclotricatechylene; Porph–PIM- Porphyrin-PIM; Trip–PIM- Triptycene-PIM; and HATN–PIM- Hexaazatrinaphthylene-PIM).

Adsorbent materials have been researched for their exceptional potential to match and surpass the storage capacities of a conventional physical storage system. The materials for hydrogen storage are basically classified according to their modes of absorption. Fundamentally, it is defined as the nature of the interaction between the hydrogen gas molecules and the material(s) of the storage system. There have been a number of different materials researched in the recent years in a direction to achieve an appropriate storage capacity. The materials are classified into two main categories of storage mechanism, *viz*., chemisorption and physisorption. In chemisorption, the hydrogen atoms interact chemically with the storage material via chemical bonds. In this category, metal hydrides, intermetallic hydrides and complex hydrides are included. In physisorption, the hydrogen gas molecules are adsorbed on to the surface of the storage material by weak physical interaction, e.g., van der Waals, electrostatic, dipole–dipole moment, dipole-induced dipole, etc. In this category, porous sorbent materials like metal organic frameworks (MOFs), nanoporous carbon materials, carbon nanotubes (CNTs) which include both, single-walled CNTs and multi-walled CNTs, and polymers. The latter has two main categories, *viz*., (a) non-conducting polymers and (b) conducting polymers. The non-conducting polymers include polymers of intrinsic microporosity or PIMs, e.g., cyclotricatechylene-incorporated PIM (CTS–PIM), porphyrin–PIM (Porph–PIM), triptycene–PIM (Trip–PIM), Hexaazatrinaphthylene–PIM (HATN–PIM); and hyper-crosslinked polymers (HCPs). The HCPs include Cooper’s HCP. Conducting polymers include polyaniline (PANI), polypyrrole (Ppy), polythiophene, poly (paraphenylenevinylene), poly (paraphenylene), and polyacetylene. A summary of different hydrogen storage materials is shown in Figure 2.

## 3. Conducting Polymers for Hydrogen Storage: Molecular Structure and Electrical Properties

**Figure 3:**
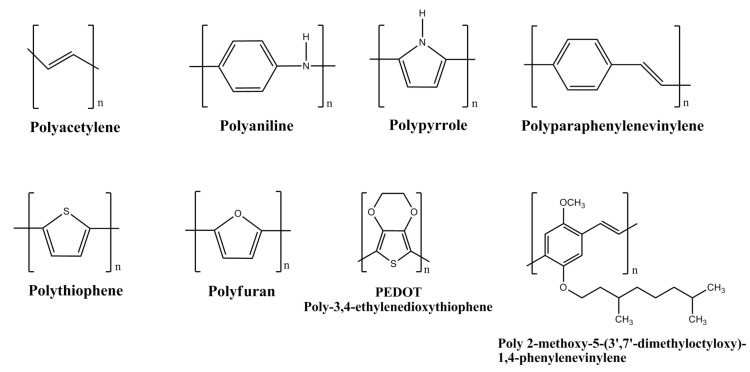
Molecular structures of conducting polymers.

Conducting polymers are also known as intrinsically conducting polymers (ICPs). They have unique electronic properties attributed to the unhybridized *p*-orbitals with an unpaired electron or π-electron at each sp^2^ hybridized carbon atom in the molecular structure. The unhybridized *p*-orbitals lie perpendicular to the aromatic ring and overlap to form an electronic cloud above and below its plane where the electrons are delocalized along the polymer chain backbone. This acts as a “highway” for the electrons to flow with high mobility along the polymeric chain and exhibit good electrical conductivity. The electrical properties of the conducting polymers are possible to be tailored and fine tuned by the addition or removal of electrons upon doping with suitable dopants. The electrical conductivity of the polymers can be enhanced up to the level of near-metal properties and often termed as synthetic metals. These may also be transformed into non-conducting or insulators by de-doping. Among the conducting polymers, polyaniline has been explored the most for hydrogen storage. The molecular structures of the conducting polymers researched for hydrogen storage are shown in Figure 3.

The application of conducting polymers for hydrogen storage was first reported by Cho et al. [8,9]. They treated the conducting polymers, *viz*., polyaniline and polypyrrole, with concentrated HCl and observed a remarkable hydrogen sorption capacity of 6 and 8 wt.%, respectively, at room temperature a under pressure of 9.3 MPa. The polymers were synthesized via a wet chemical route involving surfactants. The large dopant molecules become attached to the growing polymer chain during the synthesis process. Treating with concentrated HCl replaces ~65% of the large organic dopant molecules furnished by the surfactants, such as camphor sulfonic acid, dodecyl benzene sulfonic acid or acryl methyl propyl sulfonic acid by a substantially small moiety Cl^−^ or HCl. However, the total dopant level remained the same. This brings upon changes in the molecular as well as the overall morphological structure of the polymer and its electrical properties. The Cl^−^ doped polymer exhibited an increased capacity to hold and store hydrogen. The noteworthy observation in this experiment was the sorption property of the material. In a prior sorption process, the material was first evacuated to 10^−3^ Torr at 298 K and then allowed to absorb hydrogen. The polyaniline underwent sorption process for 1 h exhibiting a saturation at ~3.5 wt.%. The material was then projected for the second and third sorption process. Prior to the second sorption, the material was evacuated to remove the absorbed hydrogen at room temperature (RT). The second sorption attempt exhibited saturation up to 1.6 wt.% which is quite low compared with the first cycle. In the third attempt, the material was evacuated at an elevated temperature of 473 K. This treatment not only removed the previously absorbed hydrogen but also the intrinsic impurities of the polymer material which accumulate during the synthesis process. This became apparent with an enhanced absorption of hydrogen up to 6.4 wt.%. The equilibrium level of H_2_ sorption by the polyaniline were reported to be acquired in 6 h [10]. Such a longer duration required for attaining the equilibrium indicate the Knudsen diffusion of hydrogen into the pores of the polymeric material of molecular scale dimension [11]. Knudsen flow or Knudsen diffusion arises when the pore diameter of the absorbing material is smaller than the mean free path of the diffusing gas molecules. Moreover, when the density of the gas is low, the gas molecules moving into the pores tend to collide with the pore walls more frequently than with each other. On the other hand, the polypyrrole sample exhibited a sorption limit up to 1.6–2.7 wt.%. The polypyrrole sample was prepared by coating the polymer on to the polyurethane core resin. The two polymers, *viz*., polypyrrole and polyurethane, were in a ratio 1:4 [9,10].

The conducting polymers were prone to degradation at elevated temperatures. The beginning stages of the heating process during the multiple heating cycles meant for testing the absorption–desorption capacity of the polymer material involve the removal of impurities and water molecules which accumulate during the synthesis process and substantial weight losses. The subsequent heating cycles were observed to be not associated with substantial weight loss and the rate of weight reduction remained limited to ~0.5% for both polyaniline and polypyrrole samples. Furthermore, HCl treatment has been reported to induce the formation of micropores or microleaks conducive for hydrogen sorption [12,13,14]. Removal of bulky organic dopant molecules leads to the shrinkage of the pores in the bulk. The bulk conductivity of the material was observed to be reduced from 5.0 to 0.4 S/cm for polyaniline and from 3.1 × 10^−3^ to 0.9 × 10^−3^ S/cm for polypyrrole, respectively. However, on the contrary, the characteristic peak for the polyaniline in the XRD spectrum, i.e., at 2*θ* = 15°–20° was observed to be increased, indicating an improvement in the crystallinity of the material. The contradicting material characterization results were explained as increase in the intrinsic metallic properties in the microscopic regions of the polymer being restructured from the macropore dimension to micropores due to the replacement of larger organic dopant molecules by smaller chloride ions which create an increase in the amorphous properties in the bulk material [9].

### Synthesis of Conducting Polymers of Appropriate Properties

Conducting polymers can be synthesized by employing a number of methods. Polyaniline is synthesized from aniline by oxidative polymerization via different methods, *viz*., electrochemical, template synthesis, enzymatic, photochemical method, plasma vapor method, chemical synthesis, etc. The chemical route can be further carried out in a number of ways, *viz*., heterophase synthesis, interfacial synthesis, metathesis, self-assembly, seeding, solution phase synthesis, sonochemical synthesis, etc.

**Figure 4:**
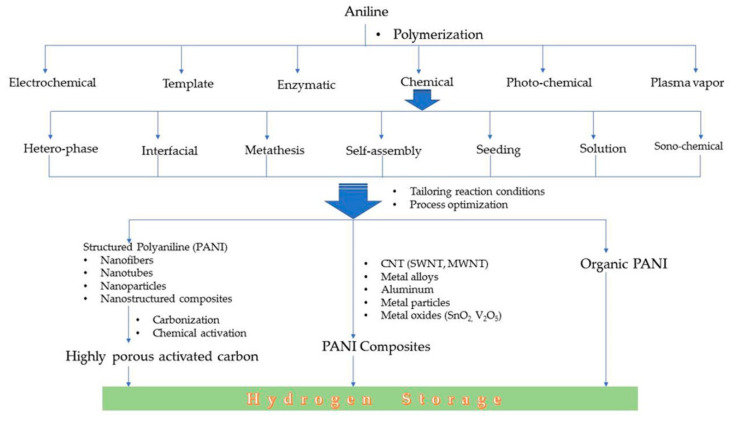
Different methods of the synthesis of polyaniline from aniline monomer.

**Figure 5:**
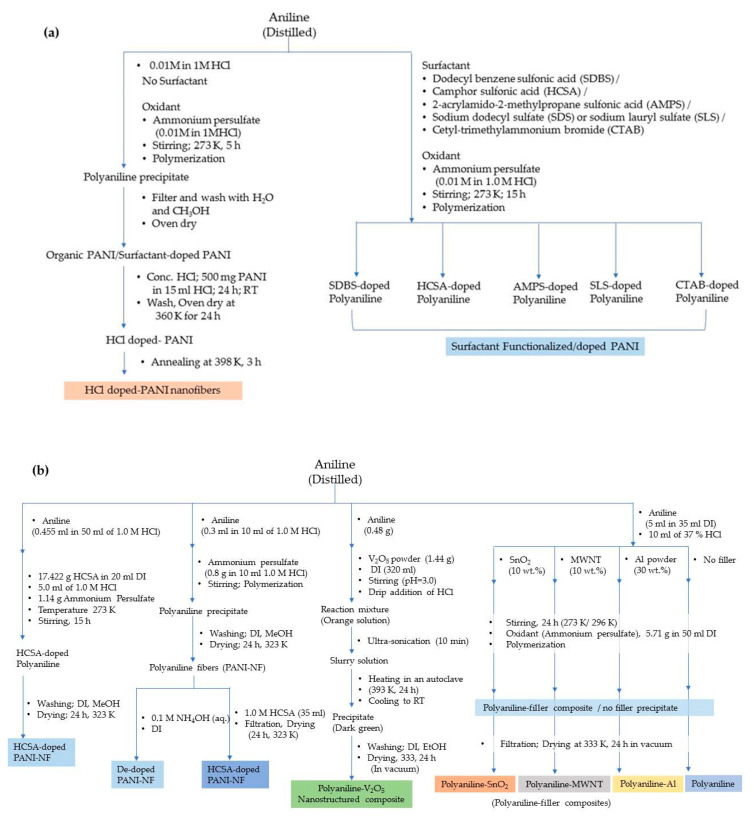
Synthesis of (**a**) polyaniline and polyaniline nanofibers in the presence/absence of surfactant, and (**b**) polyaniline composites with nanostructured particles (RT-Room temperature; DI-Deionized Water).

The different standalone polyaniline structures explored for hydrogen storage are nanoporous polymeric structure, one-dimensional nanofiber structure, nanoporous polymer with hyper-crosslinked structure, etc. The nanostructured and porous polymeric structure is an efficient architecture for a faster ion/charge transport as well as a provider of ample space to adsorb and store hydrogen. It is possible to control the diameter and structural dimensions of polyaniline nanostructures by fine tuning the synthesis parameters. A summary of the synthesis methods employed for polyaniline is displayed in Figure 4 and Figure 5.

**Figure 6:**
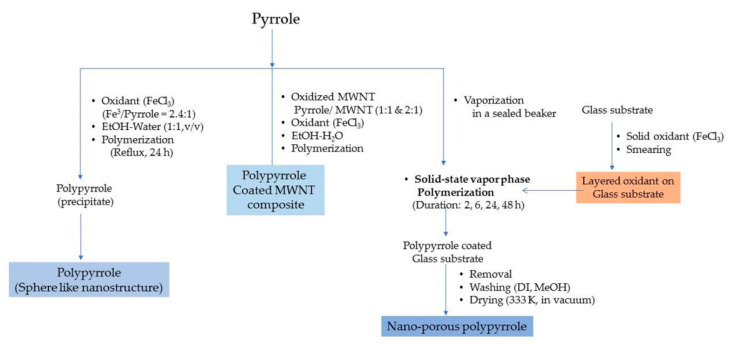
Synthesis of polypyrrole using wet chemical and solid state vapor phase polymerization methods.

**Figure 7:**
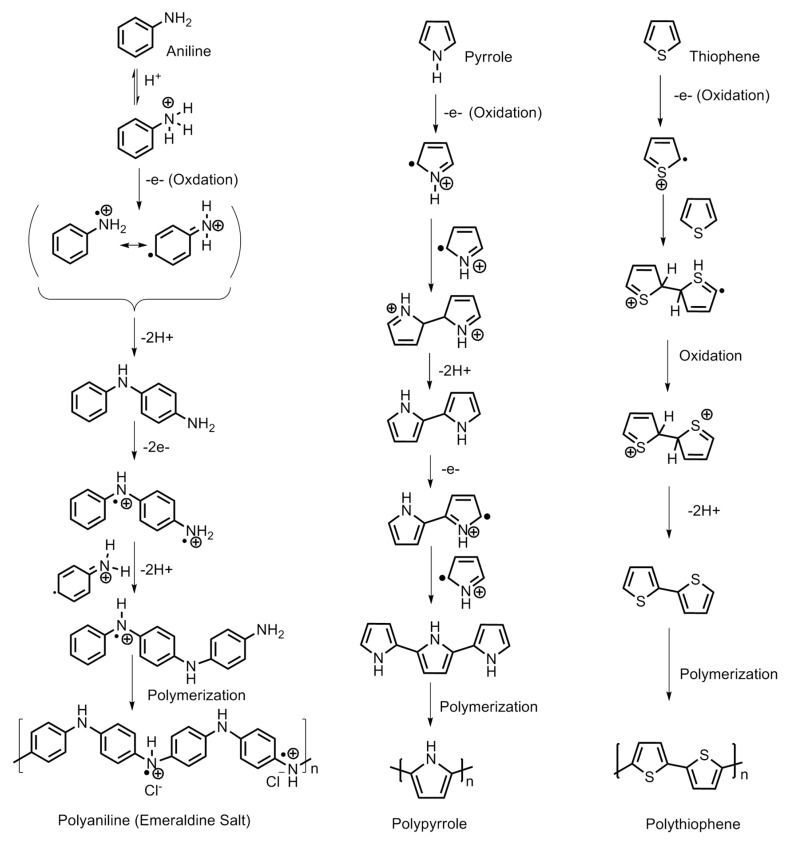
Steps involved in the synthesis of polyaniline, polypyrrole and polythiophene.

Polypyrrole is known for its remarkable electronic conductivity and excellent thermal stability. Furthermore, it is relatively convenient to synthesize by electrochemical polymerization and chemical oxidative polymerization. The electrochemical route of synthesis gives rise to a highly controlled morphology and microstructure of polypyrrole and the direct development of its polymer structure. On the other hand, the chemical route is capable of producing polymer in large amounts and ready for direct application. The physico-chemical and electrical properties are largely dependent on the type of oxidant employed and the reaction parameters, *viz*., the concentration ratio of the monomer and oxidant, temperature and reaction duration. Attia et al. developed nanoporous polypyrrole with a sponge-like structure via solid-state vapor-phase polymerization method with a purpose to induce controlled pore formation. They synthesized polypyrrole employing a method in which the substrate was exposed to pyrrole vapors for different durations in a sealed beaker. The steps are shown in Figure 6. They concluded that longer exposure durations did form a compact polymer with a high thermal stability but poorer porosity compared with the product which was obtained after a shorter duration reaction process. The latter created nanoporous material appropriate for hydrogen absorption but exhibited a lower thermal stability [1]. The steps involved in the oxidative polymerization of aniline, pyrrole and thiophene are shown in Figure 7.

The material exhibited the maximum reversible hydrogen adsorption capacity of 2.2 wt.% at 77 K at 9 MPa and the average isosteric heat of adsorption recorded was 7.51 kJ/mol. During the washing process, the unreacted solid oxidant particles were dissolved off creating internal pores and pockets as well as on the surface, giving rise to a sponge-like structure. The samples showed thermal stability up to 423 K and demonstrated a two-step mass loss process, *viz*., below 373 K owing to the elimination of water and 443 K owing to the loss of dopant molecules. At 573 K, there was recorded a sharp mass loss indicating the decomposition of polymer chains. [1]. A different study conducted by Germain et al. revealed a hydrogen absorption capacity of 1.6 wt.% at 77 K and 0.4 MPa by nanoporous pyrrole synthesized by hyper-crosslinking the polypyrrole chains through complex chemical reactions [15].

## 4. Mechanism of Hydrogen Storage

**Figure 8:**
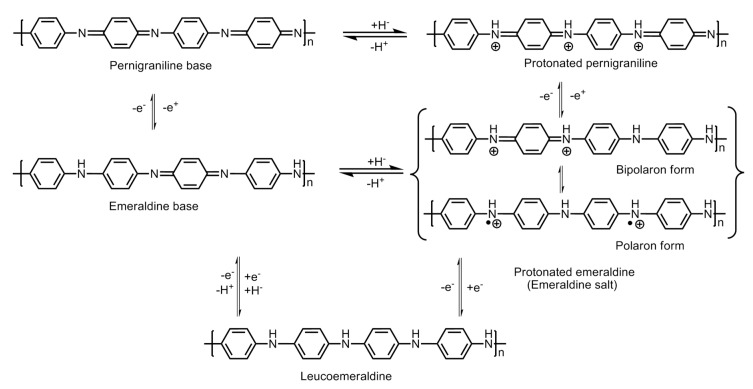
Molecular structures of the different redox forms of polyaniline.

Polyaniline is known to exist in three different oxidation states with specific molecular structures, namely the emeraldine base which is a half oxidized/half reduced state; the leucoemeraldine base, which is a fully reduced state; and the pernigraniline base, which is a fully oxidized state. The imine groups in the emeraldine base, when protonated using acids, give rise to emeraldine salt (Figure 8). Protonation results in the rearrangement of the polymer structure from an intermediate bipolaron to a polaron structure which conducts in nature.

**Figure 9:**
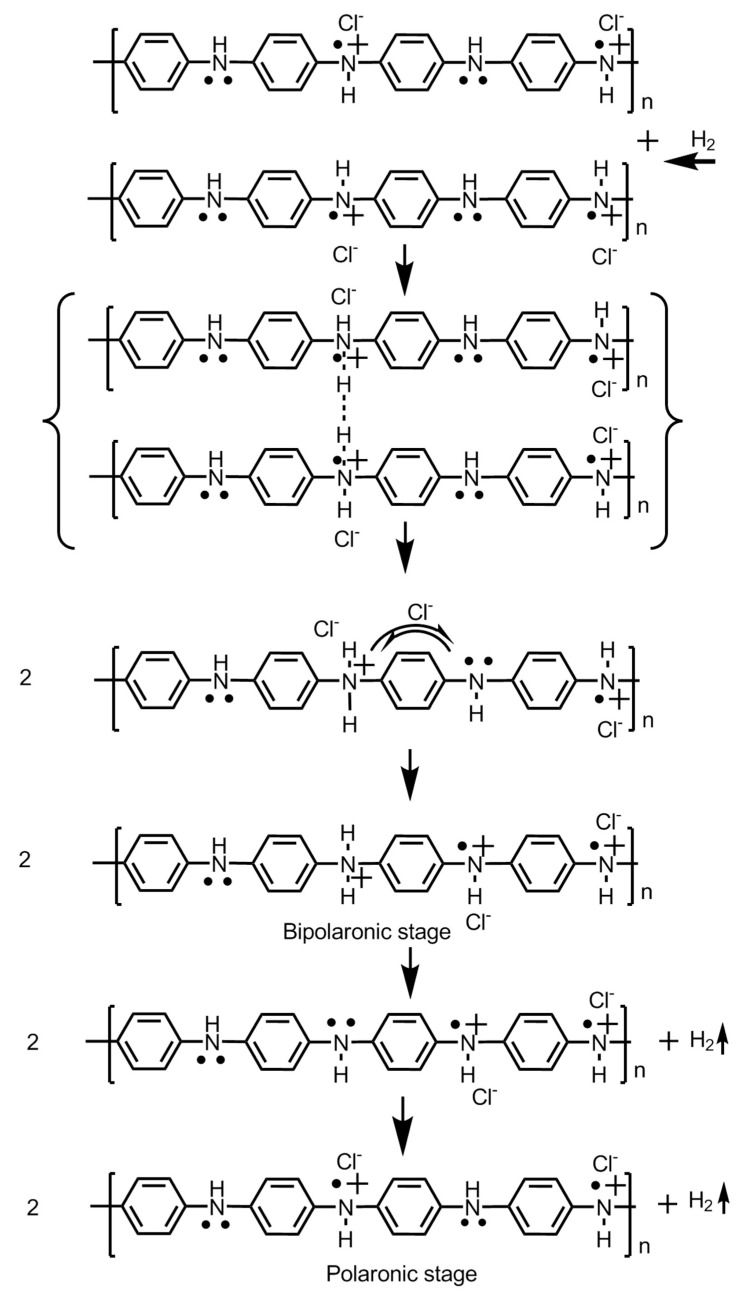
Steps involved in the process of hydrogen adsorption and storage.

The hydrogen absorption capacity is largely dependent upon the morphology, especially the porosity of the polymer material. It is also attributed to the extended π-electron system which plays an important role in improving the interaction between the hydrogen gas and polypyrrole material. The steps involved in the adsorption of hydrogen in polyaniline are shown in Figure 9. Attia et al. conducted another work involving polyaniline–polypyrrole composite material and investigated the hydrogen absorption dynamics. They synthesized the composite by coating the polyaniline nanofibers with a thin layer of polypyrrole via the process of vapor phase polymerization and reported a two-fold increase in hydrogen storage capacity. The standalone polyaniline nanofibers exhibited an absorption of 0.46 wt.%, whereas in combination as a composite, it exhibited an absorption of 0.91 wt.% of hydrogen gas. This indicates a superior property of the polymers when employed as a composite [16]. For the interaction of hydrogen gas atoms with doped polyaniline, the three cases are explained below [2,17,18].

(a) When polyaniline is doped with Cl^−^:

The amine groups of the polymer chain are charged. The hydrogen gas atoms approach the charged amine groups and dissociate to form new N–H bonds with the amine of the polymer chain.

Following this charge transfer between amine groups located in the vicinity of the polymer molecule and neutral ammonium becomes unstable and polyaniline decomposes to a polaronic doped state. When the hydrogens are released, the process is reversible.

(b) When polyaniline is de-doped or undoped:

The emeraldine base contains amine and imine nitrogen atoms at alternate positions forming the polymer, an insulating material and charge-transfer is blocked. As a result, the hydrogen fails to interact with the polyaniline chains and no absorption takes place. Hence, the doping of the polyaniline is crucial for hydrogen gas absorption.

(c) When polyaniline is protonated/deprotonated

The protonation of the polyaniline introduces quaternary ammonium groups which repel the incoming hydrogen molecules to adsorb on the surface. Aniline has electron donating properties. Upon protonation, the quaternary ammonium groups becoming electron withdrawing and consequently, the ability of the polyaniline material to adsorb hydrogen decreases.

## 5. Role of Dopants and Physico-Chemical Treatment

### 5.1. Role of Surfactants

**Figure 10:**
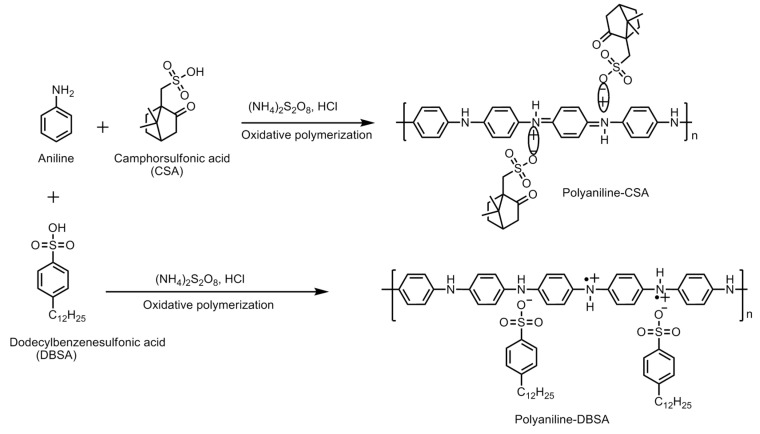
Schematic representing the effect of surfactant employed during the synthesis of polyaniline. The surfactant molecules become incorporated into the polymer structure and decide its morphology significantly.

In order to achieve the desired morphology of the polymer with appropriate properties, a number of dopants were added to the composition. These dopants may be surfactants, metallic/metal alloy/metal nanoparticles, crosslinking agents, different polymeric molecules, etc. The surfactants are known to impart their unique contribution to the growing polymer chain and decide the final architecture. Polyaniline has been explored extensively by fine tuning the amount of surfactant molecules and the reaction parameters during the synthesis process and create versatile morphologies. For example, sucrose, when added as a surfactant to the reaction mixture, act as “pillars” between the growing polyaniline nanofiber layers, creating porous spaces for the hydrogen molecule to absorb efficiently and facilitate its storage [19]. A schematic representing the effect of the surfactant is employed during the synthesis of polyaniline, as shown in Figure 10. The surfactant molecules are incorporated into the polymer structure and decide its morphology significantly.

**Figure 11:**
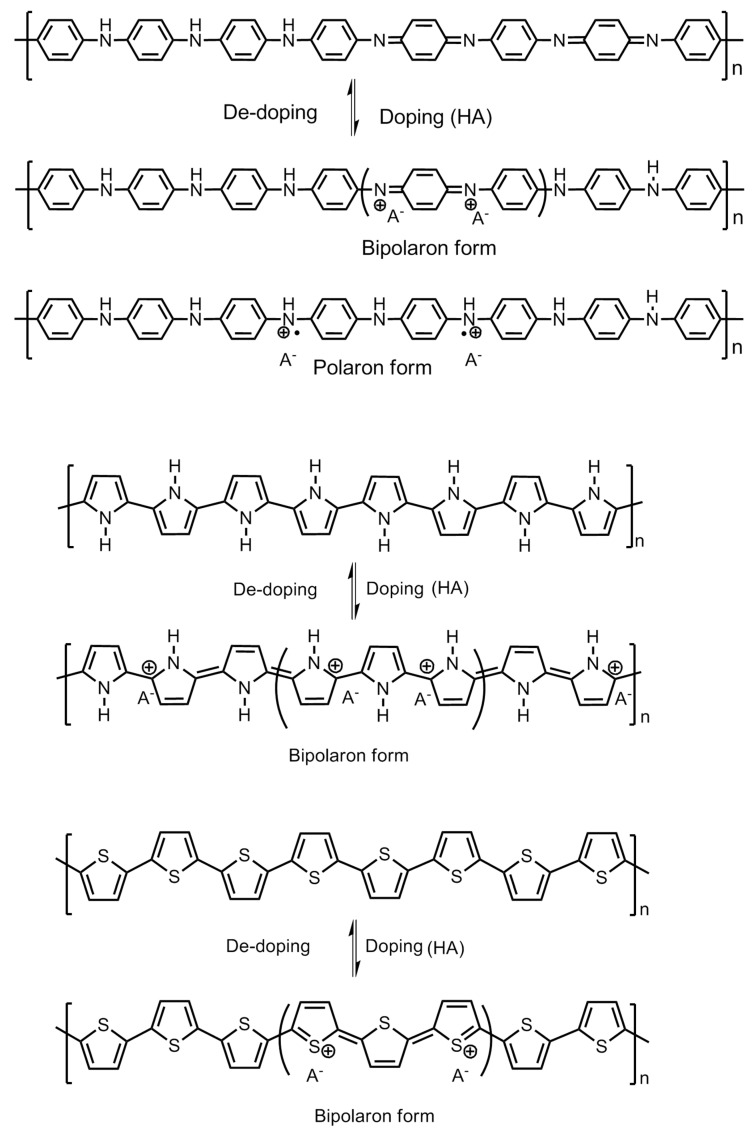
Mechanism of doping–de-doping in polyaniline, polypyrrole and polythiophene.

As it become apparent that the undoped or de-doped polyaniline cannot take part in a hydrogen adsorption process owing to its co-conductive properties, a schematic representing the electronic properties of polyaniline, polypyrrole and polythiophene is summarized in Figure 11. The role of the dopant molecule is crucial for hydrogen adsorption. The common dopants for the conducting polymer are hydrochloric acid (HCl), camphor sulfonic acid (HCSA), dodecyl benzene sulfonic acid (DBSA), acryl methyl propyl sulfonic acid, etc. When these dopants are incorporated into the polymer chain molecule, they impart steric hindrance of little to substantial dimensions to the approaching hydrogen gas atoms at the amine sites. Among these dopant molecules, HCl is a dopant of smaller size, whereas the rest are comparatively quite larger in size. The Cl^−^ anion group has a radius of 0.181 nm whereas the radius of -CSA is 0.307 nm. The Cl^−^ anion on the HCl-doped polyaniline imparts a lower steric hindrance compared with the -CSA anions as dopants. A lower steric hindrance around the charged nitrogen sites on the polyaniline chain allows larger room for the approaching hydrogens to form N–H bonds compared with the larger and bulky dopant molecules, which hinders the process substantially. In an interesting experiment, Attia et al. incorporated an additional layer of polypyrrole into the surface of polyaniline chains. The molecular interactions of polypyrrole with polyaniline nanofibers give rise to a higher steric hindrance around the nitrogen sites. This results in reduced opportunities for the approaching hydrogen towards the changed nitrogen of amine groups on the polymer chain. If the polyaniline chains are already doped with large and bulky -CSA dopants, the absorption of hydrogen is greatly reduced. They further reported a lower storage capacity of 0.52 wt.% for the HCSA-doped polyaniline nanofiber composite with polypyrrole, compared with 0.91 wt.% for the HCl doped composite synthesized by same chemical process [16].

The synthesis of polyaniline using the template method employing electrospinning of the polymer solution delivers thermally stable polyaniline and has been shown to store greater amounts of hydrogen at an elevated temperature. The electrospun fibers have shown swelling and shortening during hydrogen adsorption. Phani et al. synthesized polyaniline by electrospinning of polyaniline–polyvinyl alcohol with polyaniline amount of 15 wt.% and obtained a hydrogen storage capacity of 3 wt.% at 323 K/8 MPa, and 6–8 wt.% at 373 K/8 MPa. Electrospinning creates continuous and aligned fibers of nanoscale diameter under high electric field. The spinning under high electrical field effect leads to the development of a jet arising from repulsion forces of a charged solution overcoming the surface tension of the solution under a high electrostatic field [20]. Srinivasan et al. obtained electrospun fibers directly from the polymer solution and observed the hydrogen storage capacity of 3–10 wt.% at 298–398 K [21].

### 5.2. Role of Metallic/Metal Alloy/Metal Oxide Nanoparticle Dopants

**Figure 12:**
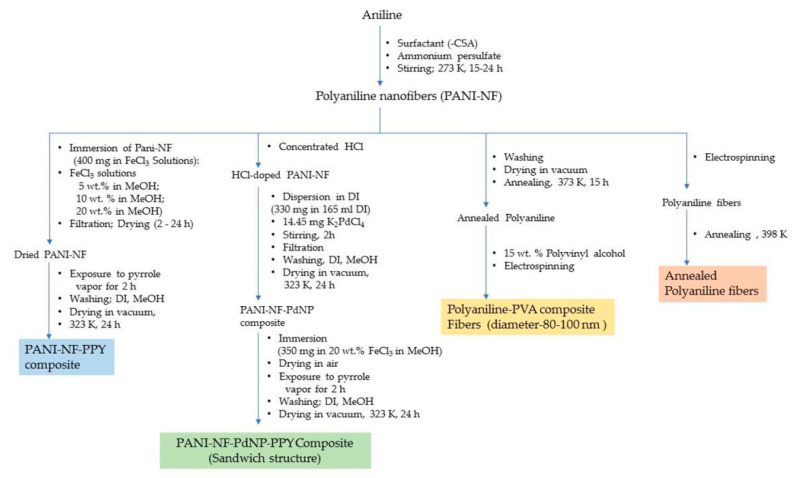
Steps involved in the synthesis of different polyaniline composite. (PVA-Polyvinyl Alcohol).

Incorporation of nanostructured fillers in the polyaniline structure, such as carbon nanotubes (CNTs), including both single walled (SWNTs) and multi-walled carbon nanotubes (MWNTs), SnO_2_, aluminum powder, V_2_O_5_, etc., has been investigated and found to act as a catalyst in hydrogen adsorption and storage. However, the results are not positive in every case. For example, the standalone polyaniline showed a storage capacity of 0.35 wt.% at 398 K/6 MPa whereas the composites incorporating MWNTs and SnO_2_ did not show any improvement at same testing parameters. However, at an elevated temperature, an increase in hydrogen adsorption has been recorded [22]. In another study, polyaniline in a composite structure with different dopants like boric acid (PANI-B), boron trifluoride (PANI-BF_3_) and their corresponding CNT composites, *viz*., PANI-B-SWNTs-10%, PANI-BF_3_-SWNTs-10%, PANI-B-MWNTs-10% and PANI-BF_3_-MWNTs-10%, tested at 77 K/10 MPa, showed an increase in hydrogen adsorption with an increase in pressure and an enhanced storage capacity. SWNTs incorporated in the composite structure have been observed to outperform the standalone CNTs, while boron and boron trifluoride contributed in imparting a molecular sieving property and electronic conductivity which result in the instability of hydrogen storage [23]. Polyaniline in a composite structure with V_2_O_5_, i.e., PANI-V_2_O_5_ tested at 77 K/7 MPa showed hydrogen adsorption capacity of 1.8 wt.% whereas standalone polyaniline showed an adsorption capacity of 0.15 wt.%. PANI-V_2_O_5_ tested at 298 K/9 MPa showed a lower adsorption of 0.16 wt.%. V_2_O_5_ contributes to the intercalation to the polymer architectures where it sits between the layers or interlayer spaces creating narrow spaces of dimension ranging between 1.1 and 0.72 nm, which is a preferable size room for hydrogen to enter conveniently, become adsorbed and stored [24]. V_2_O_5_ also facilitates the integration of other polymer chains having conjugated double bonds, such as polyaniline and polythiophene in the composite structure, creating an additional intercalating structure to the parent polyaniline architecture. The reaction steps for the synthesis of polyaniline nanofibers incorporating nanostructured particles are summarized in Figure 5. This hybrid structure shows a hydrogen adsorption of 3.3 wt.% at 473 K/8.2 MPa. Further doping with nickel nanoparticles to the composite structure (V_2_O_5_-PANI-PTH-Ni) showed an improved adsorption capacity at a lower pressure of 7.5 MPa, but remained unchanged in comparison with the undoped Ni counterpart at the previous test conditions. Nickel is believed to enhance the uptake at lower pressure. The combined effects of both the polymer materials, *viz*., polyaniline and polypyrrole, in the composite structure along with intercalating V_2_O_5_ and Ni creating spaces between the polymeric layers, is believed to enhance the adsorption capacity due to the overall increased surface area of the polymer providing the availability of extended lengths of conjugated π-electrons along the polymer chains [25]. On the other hand, doping polyaniline with titanium was found to show a high adsorption of hydrogen theoretically. However, experimental results disapprove the hypothesis which was attributed to clustering of the nanoparticles. Doping composite polymers, e.g., polyaniline nanofibers and polypyrrole composites with palladium nanoparticles of an average diameter ranging between 4 and 5 nm, where the nanoparticles are embedded between the layers of the two different polymeric materials in a sandwich-like morphology, is represented in Figure 12. This shows a hydrogen storage capacity of 0.12 wt.% which is lower than the standalone counterparts [16]. The role of transition metal nanoparticles in the polymer composite structure facilitates the dissociative chemisorption. The hydrogen molecules as hydrogen atoms are temporarily associated on the surface of metal nanoparticles acting as catalysts which later migrate to the surface of the support material. The latter is largely responsible for the adsorption and storage of hydrogen. Polyaniline in a composite structure with AB_2_ and AB_3_ type alloys has been shown to improve the hydrogen storage capacity in terms of adsorption kinetics. The composite structures have been observed to attain the adsorption equilibrium in a shorter duration of time indicating improved rates of adsorption. This is attributed to the participation of the free radicals present in the polyaniline chain interacting with hydrogen molecules facilitated by AB_2_–H/AB_3_–H bond formation. Similar reasons are believed to be applicable in the case of AB_5_–H type alloys as well.

### 5.3. Role of Crosslinking Agents

Polyaniline has been synthesized in a number of ways to achieve the porosity of desired dimensions, *viz*., microporous (pore diameter ≥ 75 µm), mesoporous (pore diameter 2–75 µm), microporous (pore diameter ≤ 2 µm), and nanoporous structures (pore diameter of 100 nm and less). The nanoporous polyaniline structures have been observed to adsorb and store hydrogen efficiently due to their high surface area. In recent years, a number of efforts have been focused towards tailoring polyaniline nanostructured structures and their composites with other conducting polymers, as well as metal nanoparticles to achieve highly efficient hydrogen gas storage material.

**Figure 13:**
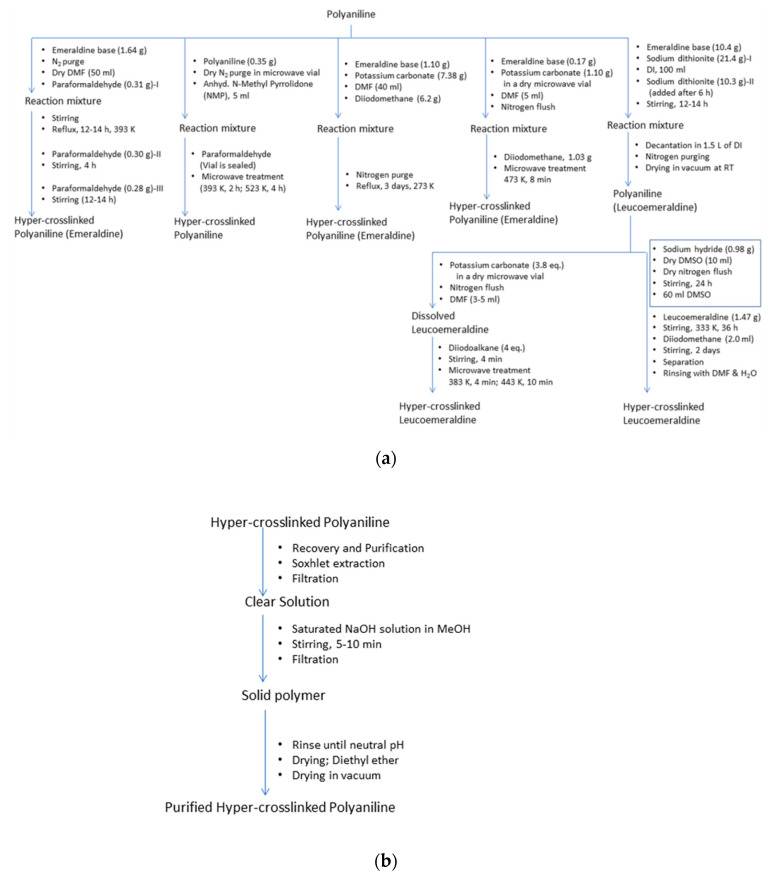
(**a**) Hyper-crosslinking, (**b**) recovery and purification of polyaniline. Artwork developed from descriptions in the Ref. [26].

**Figure 14:**
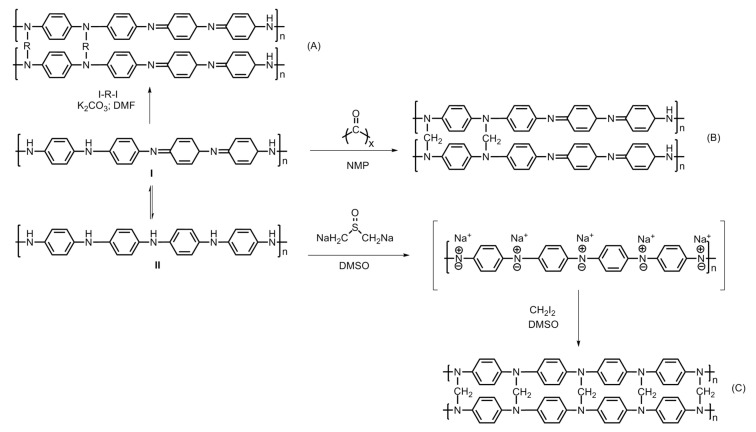
Steps involved in the hyper-crosslinking of polyaniline; (**A**) diiodoalkanes (single-step crosslinking), (**B**) paraformaldehyde (three-step crosslinking) and (**C**) dimethylsulfoxide (two-step crosslinking). Adapted from Ref. [26].

**Figure 15:**
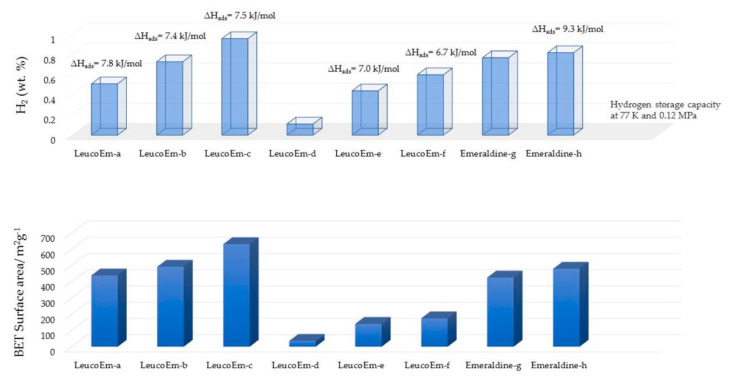
A comparison of results among hyper-crosslinked polyaniline materials obtained from different crosslinking agents and methods of preparation. Leucoemeraldine (LeucoEm)-a,b,c: hyper-crosslinked with diiodomethane in DMF using microwave-assisted process (concentration of polyaniline in a: 0.038 g mL^−1^); b: 0.058 g mL^−1^; and c: 0.078 g mL^−1^)): Leucoemeraldine-d: hyper-crosslinked with DMSO assisted diiodomethane using conventional process; Leucoemeraldine-e,f: hyper-crosslinked with paraformaldehyde (equivalents of paraformaldehyde relative to the amine content of polyaniline in e: 3.7 and f: 2.4) using microwave assisted process; Emeraldine-g: hyper-crosslinked with diiodomethane using conventional process; Emeraldine-h: hyper-crosslinked with paraformaldehyde (equivalents of paraformaldehyde relative to the amine content of polyaniline: 2.4) using the microwave-assisted process. Artwork developed from description given in the Ref. [26].

One of the popular methods for obtaining nanoporous polyaniline structure is hyper-crosslinking agents, e.g., diiodoalkanes (single-step crosslinking), dimethylsulfoxide (two-step crosslinking) and paraformaldehyde (three-step crosslinking). Germain et al. carried out the hyper-crosslinking of polymer chains via both methods, *viz*., conventional synthesis and microwave assisted synthesis (Figure 13 and Figure 14) [26]. Hyper-crosslinking between the polymer chains facilitates efficient charge delocalization and the reduction of the charge density on the nitrogen atom of the amino groups. The crosslinking prevents the pores from collapsing when the solvent is removed as well as degasification during the adsorption–desorption cycles. The aromatic ring present in the polyaniline molecular structure is believed to be the main site for hydrogen adsorption [27,28,29]. Additional electron donating groups to this aromatic ring further increases the possibility of hydrogen bondage and storage capacity. On the other hand, electron withdrawing groups decrease the hydrogen storage. A study conducted by Germain et al. reported on de-doped and hyper-crosslinked polyaniline, showing an enhanced hydrogen storage capacity compared with its protonated polyaniline counterparts. This is attributed to the high surface area of the polymer structure. The hyper-crosslinked polymers showed a hydrogen storage capacity of 2.2 wt.% at 77 K/3.0 MPa and high enthalpies of adsorption of 9.3 kJ/mol rendering the process exothermic. The de-protonated hyper-crosslinked polymer showed an adsorption of 0.8 wt.% at 77 K/0.12 MPa, and upon proton doping the adsorption capacity dropped down to 0.3 wt.% (Figure 15) [26]. An ideal nanoporous polyaniline-adsorbent material is hence defined as the one which possesses a high adsorption capacity for hydrogen at ambient conditions, and simultaneously, its enthalpy of adsorption should be sufficiently high to achieve the desired adsorption [26].

In this regard, most of the adsorbent materials, which include porous polymers, metal organic frameworks and porous carbon materials, have been found to exhibit low enthalpies of adsorption and therefore, show a sharp decrease in their activities. The latter is attributed to the saturation of the active sites and consequently the affinity for hydrogen adsorption decreases as the surface is covered. Because of this reason, hydrogen adsorption studies are mostly conducted at 77 K [26]. The hydrogen adsorption at room temperature has been observed to be usually less than 0.5 wt.% [18].

### 5.4. Role of Chemical Activation/Carbonization

The conducting polymers subjected to chemical activation followed by thermal treatment or carbonization give rise to highly porous nanostructured activated carbon possessing large specific surface areas with a large pore volume and potential of adsorbing large volumes of hydrogen. The properties of activated porous carbon materials are dependent to some extent on the precursor material. Chen et al. obtained activated rectangular polyaniline-based carbon tubes (ARP-CTs) via the carbonization of rectangular polyaniline tubes with hollow carbon nanospheres (HCNS; diameter 50–100 nm) as the core. The activated carbon material showed a high BET-specific surface area in the range of 1680–2415 m^2^g^−1^ and a pore volume of 1.55 cm^3^ g^−1^.

**Figure 16:**
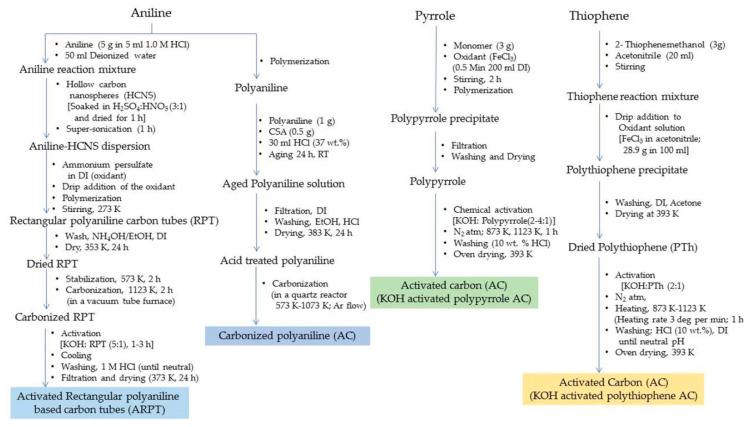
Methods of preparing activated carbons from conducting polymers.

The hydrogen storage capacity for the ARP-CTs was found to be highest for ARP-CT30 (oxidant: HCNS mass ratio of 30), i.e., 5.2 wt.% at 77 K/4 MPa. As the ratio increased from 30 to 60, the morphology changed to give rise to different storage capacities, *viz*., 3.5, 4.5 and 3.3 wt.% for ARP-CT60, ARP-CT45 and ARP-CT15, respectively. At a higher pressure of 5.5 MPa, ARP-CT30 showed a higher adsorption capacity of 8.0 wt.% at 77 K. The overall morphology of the ARP-CTs was found to be dependent upon the mass ratio between the oxidant and the HCNS [18]. Another study conducted by Sevilla et al. reported on activated carbon material obtained from KOH-activated polypyrrole. The material showed a relatively higher surface area of 3000–3500 m^2^g^−1^ and a pore volume of 2–6 cm^3^ g^−1^. The hydrogen adsorption capacity for this activated carbon material was observed to be 7.03 wt.% at 77 K/2 MPa [30]. The activated carbon material obtained from polythiophene also showed a high BET specific surface area of 3000 m^2^ g^−1^ with a pore size of 1–2.5 nm and a pore volume of 1.75 cm^3^ g^−1^. The sulfur content was estimated to be 3–12 wt.% and the hydrogen storage capacity of 5.71 wt.% at a cryogenic temperature and 2 MPa pressure [31]. The pore formation involving ferrocene for the polymerization of polyaniline followed by activation and carbonization was carried out by Chen et al. and the resultant product was iron-doped activated carbon. The decomposition of ferrocene created finite volume pore spaces in the material. The BET specific surface area of the material was 3246 m^2^ g^−1^ and the pore volume 2.06 cm^3^g^−1^. The material exhibited a hydrogen storage capacity from 5.3 to 6.2 wt.% at 77 K/5 MPa and 0.6 to 0.88 wt.% at 293 K/8 MPa [32]. In a different study, Hwang et al. obtained activated carbon materials with different amounts of carbon loading from acid-treated polyaniline and recorded a storage capacity of 0.27 wt.% at 223 K/2 MPa, 1.09 wt.% at 223 K/5 MPa and 0.35 wt.% at 223 K/7 MPa for the material with a carbon loading of 68.5%. The activated carbon with 74.1% loading showed a storage capacity of 4.0 wt.% at 173 K/7 MPa [33]. A schematic displaying the steps in the preparation of activated carbons from conducting polymers are summarized in Figure 16.

## 6. Conducting Polymer for Hydrogen Fuel Cell

### 6.1. Early Stages of Conducting Polymer Membrane for Hydrogen Fuel Cell

The idea of solid electrolyte membranes for fuel cells was introduced by Grubb in early 1960 [34] by using poly (phenolformaldehyde sulfonic acid) resin. This was the first polymer used as an electrolyte membrane (PEM) in a fuel cell. These PEMs were later found to be unsuitable because of their weak hydrolytic stability, short lifetimes (100 h at 323.15 K), less efficiency and poor mechanical strength. Therefore, these failed to meet the requirements for the proper working of a fuel cell for a significant duration. Relatively more durable polymeric materials, namely, sulfonated polymer membranes (Polystyrene sulfonic acid membrane) with improved properties (capable of functioning for 200 h at 333.15 K) were developed by crosslinking styrene–divinylbenzene into an inert fluorocarbon matrix. The stabilities of the sulfonated polymer material under both wet and dry conditions made it suitable for NASA Gemini space missions during the period 1962–1964. These membranes achieved considerable success as compared with phenol–formaldehyde sulfonic acid, in terms of both efficiency as well as lifetime. However, these PEMs were not found to be suitable for commercial purposes, because of the need of a greater lifetime and high oxidative stability. The polytrifluorostyrene sulfonic acid polymeric membranes developed in the later years had been reported to exhibit an increased efficiency up to 70 mWcm^−2^ and a lifetime up to 8000 h. However, their poor mechanical properties made them inappropriate for practical use in commercial fuel cell applications. The progress in material development in recent years through overcoming a range of challenges at various levels is discussed in this section.

### 6.2. Poly (Perflurosulfonic Acid) (PFSA) Nafion-Type Membranes

**Figure 17:**
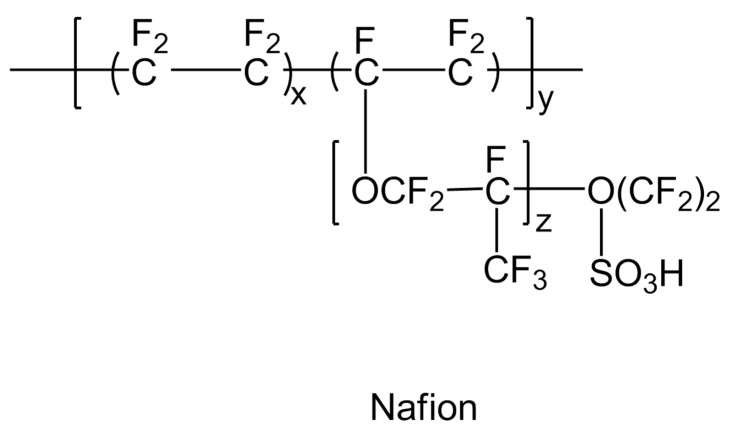
Polyperfluorosulfonic acid (PFSA) (Nafion)-type membrane.

A major breakthrough occurred in 1959 when perflurosulfonic acid (PFSA) was discovered as a durable (lifetime of several thousands of working hours below 373.15 K) and an efficient polymer membrane material with significant proton conduction as well as gas barrier properties for fuel cells [5]. This is a combination of hydrophobic Teflon as a main chain and hydrophilic sulfonic acid as a side chain. The molecular structure of polyperfluorosulfonic acid (PFSA) (Nafion)-type membrane is shown in Figure 17. Hence, membrane certain degree of hydration in the polymer membrane is required for the smooth conduction of protons. The thickness of the PFSA membrane is one of the main deciding factors for any desired application. The thinner membrane of PFSA is found to be more suitable for hydrogen/air applications to minimize ohmic losses. The stability and performance of the PFSA membrane also depends on the length of the side chain. One of the best examples of long side chain polymeric material under this category is Nafion, which became very popular due to its high proton conductivity, moderate water swelling, ability to bear chemical attacks and low release rate of degradation products into the surrounding medium inside the fuel cell [35]. Hence, Nafion and Nafion-like sulfonated fluorocarbon polymer members are considered to be revolutionary and pioneer materials for the commercial and industrial applications of fuel cells around the world. Nafion works perfectly under hydrated conditions and it carries out proton conduction through water filled channels. The sulfonic groups present in the Nafion structure are hydrophilic in nature and enable the molecule to absorb moisture. Thus, Nafion with low water content features tiny isolated water clusters within the molecule and acts like an insulator. As the moisture content in the molecule increases, these tiny droplets connect to form channels or random networks, also called percolation channels, and facilitates proton conduction. The Nafion molecule, fully saturated with moisture, exhibits proton conductivity values close to the conductivity values of bulk electrolyte membranes.

In spite of numerous benefits like excellent proton conductivity even at high operating temperatures up to 463.15 K, the perflurosulfonic acid-based membranes face several challenges, *viz*., (a) inoperability at high temperatures; (b) high cost of materials; (c) need humidification equipment to reach the required level of humidity; (d) waste products are harmful to the environment; (e) swelling and shrinking because of changes during water uptake; (f) mechanical degradation owing to long duration thermal and humidity cycling; and (g) chemical degradation. These challenges have become a driving force for the development of new affordable polymer electrolytes without compromising its qualities [7,36].

### 6.3. Partially Fluorinated Membrane

**Figure 18:**
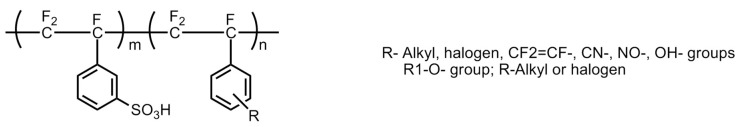
Basic molecular structure of the sulfonated poly (*α*,*β*,*β*-trifluorostyrene) BAM3G (Basic Advanced Materials 3rd Generation) membrane.

To overcome the challenges of perflurosulfonic acid (PFSA) membranes, partially fluorinated membranes were proposed and developed. In partially fluorinated membranes, few microgram quantities of exotic inorganic substances, including zinc oxide, titanium oxide, and silica, were added or substituted in the PFSA polymer material to improve the physical and chemical properties of the membrane without affecting its proton conductivity. Examples of noteworthy perfluroinated materials are sulphonated *α*,*β*,*β*-trifluorostyrene; *m*-trifluoromethyl *α*,*β*,*β*-trifluorostyrene; sulphonated polymer of *α*,*β*,*β*-trifluorostyrene; and the sulphonated copolymer of *α*,*β*,*β*-trifluorostyrene; copolymer of *α*,*β*,*β*-trifluorostyrene. [37]. The basic molecular structure of the sulfonated poly (*α*,*β*,*β*-trifluorostyrene) is shown in Figure 18.

The most popular partially fluorinated membrane similar to PFSA membranes is sulfonated copolymer incorporating *α*,*β*,*β*-trifluorostyrene monomer (Basic Advanced Materials 3rd Generation, BAM3G) manufactured by Ballard Advanced Materials. This partially fluorinated membrane exhibits high proton conductivity, but the cost of this partially fluorinated membrane could not be reduced because of the presence of expansive and less available trifluorostyrene monomers. Hence, a need for a low-cost membrane with properties on par with PFSA is a main challenge for the researchers to work on to achieve adequately functioning non-fluorinated polymer membranes.

### 6.4. Non-Fluorinated Based Membranes

Non-fluorinated membranes are developed from functional polymers other than fluorinated materials.

#### 6.4.1. Polystyrene-Based Membranes

**Figure 19:**
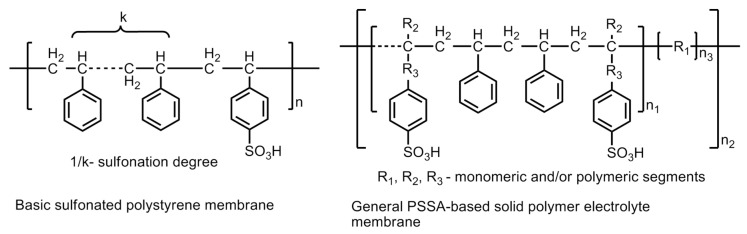
Basic structure of a polysulfonated polystyrene (PSSA) membrane and a general PSSA-based solid polymer electrolyte membrane with a probability of modification by the incorporation of monomeric, oligomeric or polymeric segments in order to achieve improved material properties.

Polystyrene materials are used to prepare low-cost polystyrene sulfonic acid -based membranes (PSSA) which have significantly reduced the cost of hydrogen fuel cells. Although, the cost is reduced, but still, these could not offer any promising device material because of instability. PSSAs were the first commercial polymer membranes developed by GE in 1955 and employed in first ever operational PEM-fuel cells in the Gemini Program. The material exhibited a very short lifetime of <200 h and high material degradation. The constituents of the membrane, e.g., ternary benzylic hydrogen and the aromatic ring protons, form a weak polymer chain, which renders it unsuitable for a satisfactory performance. The latter is because of the reduction in ion exchange capacity and conductivity. Hence, a relatively stronger chain of constituents is required to be developed in order to improve the efficiency and applicability of polystyrene-based membranes [38]. The modification of the polymeric material by substituting the ternary hydrogen atoms by methyl groups and fluorine yield a relatively stable PSSA polymer material. The basic structure of polysulfonated polystyrene (PSSA) membrane and a general PSSA-based solid polymer electrolyte membrane with the probability of modification is shown in Figure 19.

The modification of the chemical structure by incorporating different R groups of varying lengths improves the properties of the polymer backbone, or in other words, its mechanical and chemical stabilities. SSA grafted with monomers, *viz*., methacrylonitrile, acrylonitrile, methyl methacrylate or methacrylic acid and further modification by co-grafting with polyethylene–*alt*–tetrafluoroethylene (ETFE) membranes demonstrated proton conductivities up to 0.16 Scm^−1^. The styrene sulfonic acid (SSA)-oligomer grafted by copolymerization with styrene backbone chain gave rise to an enhanced conductivity up to 0.24 S cm^−1^ with improved stability under fuel cell operational conditions [39,40,41,42]. Furthermore, grafting with stable polymers like PVDF (polyvinylidine fluoride) –*grafted*–PSSA demonstrated proton conduction of 0.13 Scm^−1^ and the range including Teflon–*grafted* PSSA, (low-density polyethylene–*grafted*–PSSA, (tetra fluoroethylene–*co*–perfluoroporpylene (FEP, Teflon 100)) –*grafted*–PSSA, where, the FEP is polytetrafluoroethylene–*co*–hexafluoropropylene, demonstrated proton conductivities in the range of 0.1 × 10^−2^ to 10 × 10^−2^ Scm^−1^ [43,44,45,46]. The modification of the PSSA polymers into PSSA– (*co*–polymer) –polymers resulted in enhanced proton conductivity up to 10^−1^ Scm^−1^. Some noteworthy examples of modified co-polymeric structures are sulfonated poly(styrene–block–isobutylene–block-styrene) triblock polymers, sulfonated polystyrene–block– (ethylene-*ran*-butylene)–block-polystyrene, sulfonated styrene–ethylene copolymers, and sulfonated polystyrene (ethylene–butylene)polystyrene triblock copolymers [47,48,49,50,51,52,53].

#### 6.4.2. Sulfonated Polyimide (SPI)-Based Membranes

**Figure 20:**
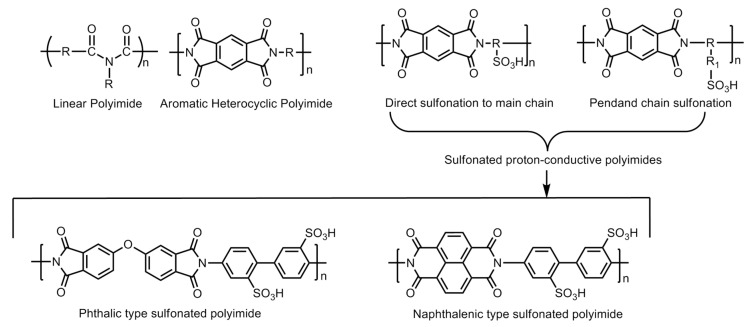
Basic molecular structures of linear polyimide, aromatic heterocyclic polyimide, Phthalic-and Naphthalenic-type sulfonated polyimides.

**Figure 21:**
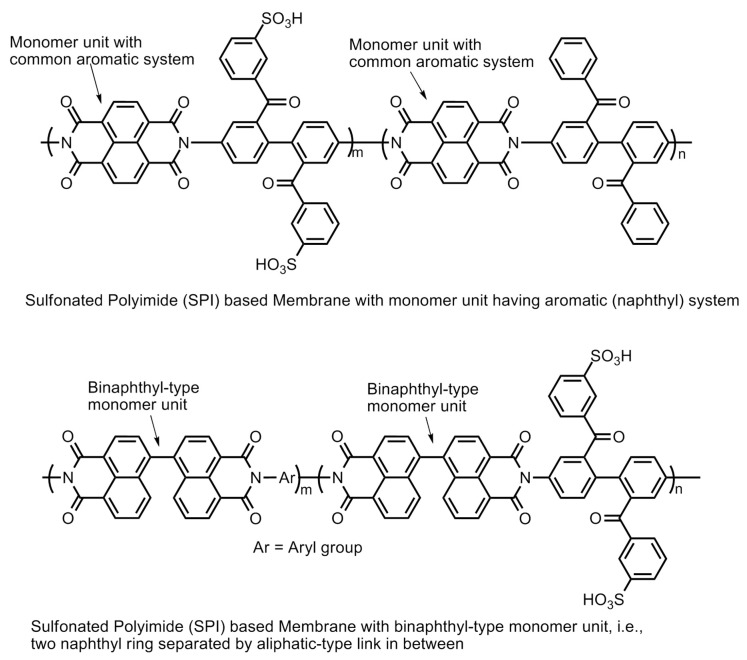
Sulfonated polyimide (SPI)-based membranes.

The polyimide family is known for less expensive polymers with excellent mechanical and thermal stability under aggressive heat and chemical treatment. However, these materials are basically insulators, but the addition of a sulfonic group to the molecular has been used to increase the hydrophilicity and proton conductivity. Hence, they are known as sulfonated polyimide (SPI) polymers. The initial conductivity of SPI-based membranes lie in the range of 0.0002–0.004 S/cm which can further improved up to 1.67 S/cm at 393.15 K and 1.20 S/cm at 353.15 K by adding graphene composite. In addition to having properties like good water uptake, swelling ratio, etc., these materials are also found to be thermally stable over the range of temperature. The main drawback of the SPI-based membrane is its instability in the hydrated state, which can be improved by the addition of the imide group. The imide group provides high resistance to the polymer towards the hydrolysis [54]. The basic molecular structure of linear polyimide, aromatic heterocyclic polyimide and their modification by introducing sulfonic acid group(s) via the direct sulfonation or pendent chain sulfonation methodologies is displayed in Figure 20. Further improvement of the polymer structures in the forms of phthalic- and naphthalenic-type sulfonated polyimides have also demonstrated the enhancement of proton conductivity. The imide molecule degradation takes place by hydrolysis. The vulnerable positions in the molecule for the nucleophilic interaction of water molecules are the carbon atoms of the neighboring carbonyl carbons. The increase in the electronic density at the carbonyl carbons by introducing a six-membered heterocyclic ring structure (i.e., naphthalenic polyimides) has been reported to enhance the stability of the polymer molecule against hydrolytic degradation (Figure 21).

The electron density of the carbons adjacent to carbonyl carbons can also be increased by introducing bulky aliphatic fragments, the incorporation of the “bridging” electron donating sulfur moiety into the parent polymer chain, the incorporation of electron donating phenoxy groups, the substitution of benzophenone groups at the *meta*-position of the imide group, and the separation of naphthyl rings by introducing an aliphatic chain or by creating a binaphthylimide configuration (Figure 21) [55,56,57,58].

#### 6.4.3. Polybenzimidazole (PBI)-Based Membranes

**Figure 22:**
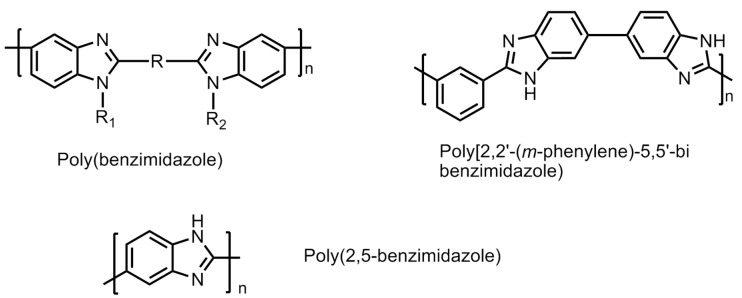
Molecular structures of some polybenzimidazole (PBI)-based membranes.

To overcome the drawbacks of low conductivity in PFSA (Nafion) membranes while operating at elevated temperatures, polybenzimidazole (PBI) membranes were designed and developed in order to find a suitable polymeric material for hydrogen fuel cell [59]. PBI is a relatively low cost, non-fluorinated rigid semi-crystalline polymer. The molecular structures of a few polybenzimidazole polymers are shown in Figure 22. It has shown good chemical resistance against chemical or oxidative degradation, thermal stability and excellent mechanical strengths. Some of the popopularly known polybenzimidazole-based polymers are phenylene-based PBI, poly(2,5 benzimidazole), and pyridine-based PBI polymers. These are studied for their high proton conductivity, particularlyat elevated temperatures (>473.15 K) under low moisture conditions. These conditions aredesirable for operating universal PEM fuel cells. Furthermore, to improve the performance of these PBI membranes, several approaches have been attempted. The main challenge with PBI-based is the presence of liquid electrolyte. The liquid is susceptible of leakage, which makes it unsuitable for application in portable devices in transport vehicles. A suitable alteration of the electrolyte material into solid form by mixing/adding/crosslinking with appropriate external agents without compromising the properties of the PBI-based membranes is under intense focus of the researchers [60].

#### 6.4.4. Polyphosphazene-Based Membranes

**Figure 23:**
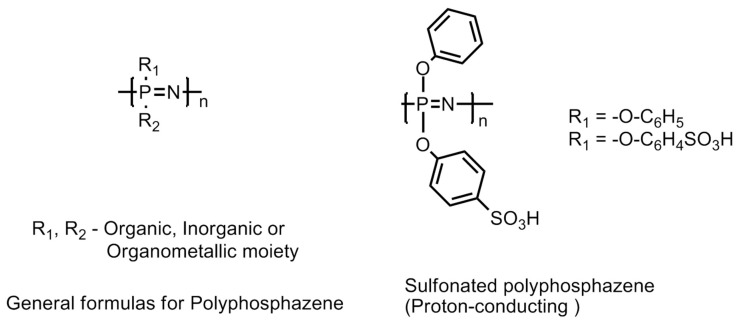
Molecular structures of polyphozphazane-based membranes.

This family of polymers is recognized by its backbone structure consisting of alternating phosphorus and nitrogen atoms as shown in Figure 23. Besides, there are also organic, inorganic or organometallic moieties attached to the phosphorus atom in the polymer backbone [61,62,63]. The [(–N = P–)] repeating units are modified by incorporating a diversified range of pending groups and give rise to many advantageous properties, such as resistance to aggressive operational conditions in the fuel cell and degradation led by free radical cleavage reactions and hydrophilicity to the polymer chain. In addition to this, the monomer unit in the polymer chain possesses high tortional mobility Functionalized polyphosphazanes, either linear or crosslinked containing hydrophilic groups, such as sulfonic acid group(s) (sulfonated-polyphosphazanes or S-Ppz), e.g., sulfonated {bis(3-methyl phenoxy)} phosphazanes have shown substantially low water diffusivity and conductivities in the range 1 × 10^−2^ to 12 × 10^−2^ Scm^−1^. The increase in the degree of sulfonation in the S-Ppz has demonstrated an increase in the proton conductivity [61,64,65,66,67,68]. The most important characteristic feature of the S-Ppz membrane is its low permeability to methanol [69]. The phosphonic functionalized Ppz (P-Ppz) polymers have been reported to exhibit lower conductivity compared with sulfonated Ppz (S-Ppz). Although, a higher degree of sulfonation imparts a higher proton conductivity but compromises the polymer properties in terms of its mechanical stability. The latter can be improved by introducing crosslinking with other polymers, e.g., polybenzimidazole and such a blend has been observed to demonstrate performance close to Nafion in terms of maximum power yield when employed in direct methanol fuel cells [61,64,70,71].

#### 6.4.5. Sulfonated Aromatic Hydrocarbon Polymer-Based Membranes

**Figure 24:**
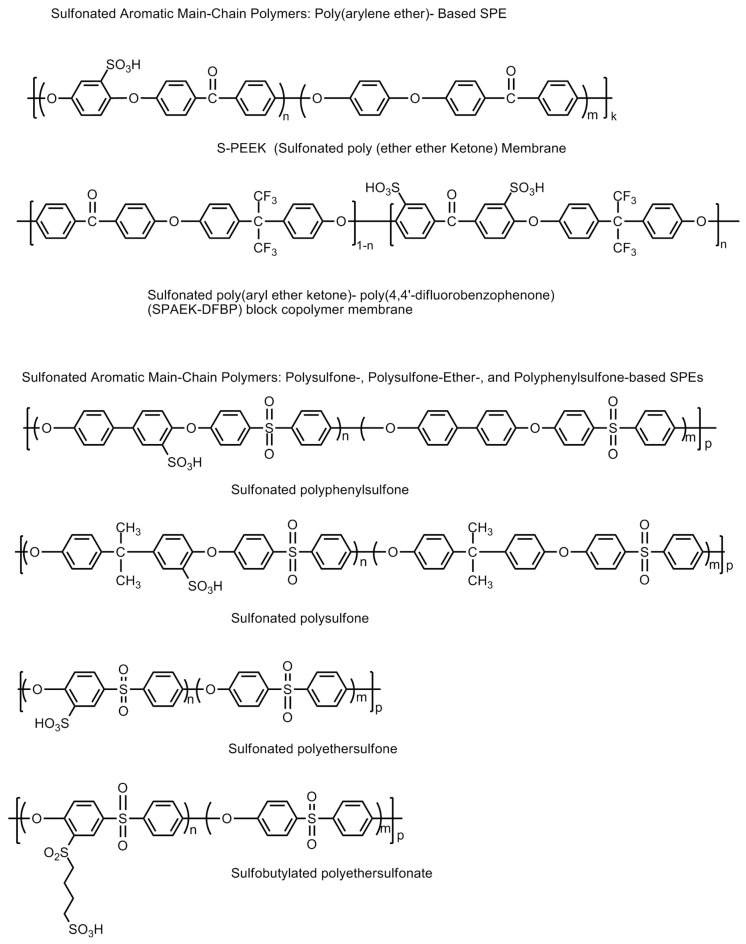
Molecular structures of sulfonated main chain polymers, *viz*., poly (arylene ether)-, polysulfone-, polysulfone (ether)-, and polyphenylsulfone-based solid polymer electrolytes.

Sulfonated aromatic polymer (SBAFs), such as sulfonated poly(arylene ether ketone)s or SPAEKs; sulfonated poly(ether ether ketone)s or SPEEKs, sulfonated poly-sulfones, sulfonated polyethersulfones, sulfonated polyphenylsulfones, sulfobutylated polyethersulfonates, etc., have been thoroughly studied and found to be suitable for fuel cell membrane [72,73]. They can be used as a replacement for Nafion because of their relatively low cost, and the easy tuning of their polymer structure to improve properties like conductivity, chemical, thermal and mechanical stability [74]. However, their chemical and mechanic stabilities are less than Nafion membranes. The molecular structures of some sulfonated aromatic hydrocarbon polymers are shown in Figure 24. A high proton conductivity and high ion exchange capacity (IEC) after the sulfonation process is reported for these materials. However, after an optimum value of the IEC, PEMs consequently lead to excessive swelling and deteriorate in their mechanical strength. Hence, a controlled designing of PEMs is always necessary to maintain water stability without excessive swelling [75,76]. The critical points of hydroxyl radical attack resulting in material degradation are the non-sulfonated phenyl ether aromatic rings on the polymer chain forming phenolic groups. The latter are vulnerable to oxidation and result in the oxidative degradation of the polymer chain or scissions [77,78]. In a quest to form suitable sulfonated aromatic hydrocarbon polymers, a lot of work has been carried out. A linear hydrophobic chain copolymer and multi-phenylated, hydrophilic domain ionomers were designed for forming proton conductive channels and maintain distinct phase separation keeping high oxidative and thermal stability to improve membrane properties [79]. The chemical and mechanical stabilities of the SPAEK polymers can be improved by fine tuning the sulfonic acid groups at appropriate positions on the polymeric backbone. When the sulfonic acid groups are attached to the pendant chains rather than the main polymer backbone, the resultant polymers have demonstrated significant stability. A high sulfonation in the polymer material (over 74%) does exhibit conductivities in the range of 0.01–0.048 Scm^−1^ at 333 K. However, higher degrees of sulfonation compromise the chemical and mechanical stabilities. This problem can be successfully addressed by introducing crosslinking of the polymeric chains. Crosslinking renders the polymer mechanically as well as chemically stable, and simultaneously saving the polymers from excessive swelling upon hydration as well as restricting the permeability towards methanol. However, excessive crosslinking has been observed to reduce the proton conductivities owing to elongation-at-breaks in the proton conduction pathways in the polymer chain [80,81,82,83,84,85,86,87,88,89,90]. SPEEK polymer electrolyte materials incorporated with SO_2_-crosslinking have been observed to demonstrate higher proton conduction up to 0.1 Scm^−1^ [84]. New fluorine-based poly(ether sulfone) polymers with clustered pendant containing sulfonic acid groups were also synthesized for high oxidative stability, good mechanical properties, and a clear phase separation of ionic domains size from 3 to 7 nm [91].

The main breakthrough was the achievement of proton conductivity comparable to PFSA in sulfonated multi-block poly(ether sulfone)s, novel poly(arylene ether)s and poly(*p*-phenylene) block copolymers. These polymers also exhibited well defined phase separation with lower water uptake and good dimensional stability [92,93,94,95]. The polymer backbone contains both hydrophilic moieties (sulfonic acid functional groups) responsible for proton conduction and hydrophobic domains (polymer backbone) responsible for mechanical and chemical stability. The incorporation of multiple co-polymer blocks into the parent SPAEK polymer chain results in a hybrid structure with additional advantages in terms of the formation of a network of hydrophilic moieties for rapid proton conduction and hydrophobic domains with substantially stronger mechanical support as well as chemical stability. The appropriate orderliness in the arrangement of hydrophobic and hydrophilic domains gives higher proton conductivity values for the polymers (0.92 × 10^−2^ and 2.09 × 10^−2^ Scm^−1^ at temperatures 333 K and 353 K) [96].

Alternately, the properties of the SPAEK polymers can be further substantially improved by (a) other polymers (e.g., polybenzimidazole, poly(ether sulfone), sulfonated/silylated polyphenylsulfone, sulfonated cyclodextrin, PVDF, epoxy resins, polystyrenesulfonic acid phosphonated polysulfone, poly(vinylidene- fluoride-co-hexafluoropropylene), phenoxy resin, polyimide, poly(ether imide), 3-aminopropyltriethoxysilane, acrylic acid-co-4-vinylimidazole, polypyrrole, polyacrylonitrile, sulfonated phenolphthalein poly(ether sulfone), poly(vinyl alcohol), sulfonated and/or silylated polyphenylsulfone); (b) inorganic fillers, such as solid heteropolyacids and their salts (e.g., H_3_PW_12_O_40_, H_4_SiW_12_O_40_, H_3_PMo_12_O_40_, Na_2_HPW_12_O_40_, CsH_2_PW_12_O_40_, H_3_SiW_12_O_40_/SiO_2_−Al_2_O_3_, Cs_2.5_H_0.5_PW_12_O_40_/Pt, H_3_PW_12_O_40_/SiO_2_, Cs_2_HPW_12_O_40_, Cs_2.5_H_0.5_PW_12_O_40_, H_3_PW_12_O_40_/molecular sieve); (c) small crosslinked polycarboxylic acid spheres (of diameter 150 nm) and polymeric phosphonic acid spheres (of diameter in submicrometer length scale); and (d) non-acid inorganic materials (e.g., zirconium phosphate sulfophenylenphosphonates, SiO_2_, ZrO_2_, TiO_2_, Zr/Ti/phosphate, Fe_3_O_4_, boron orthophosphate (BPO_4_)) [97,98,99,100,101,102,103,104,105,106,107,108,109,110,111,112,113,114,115,116,117,118,119,120,121,122,123,124,125].

**Figure 25:**
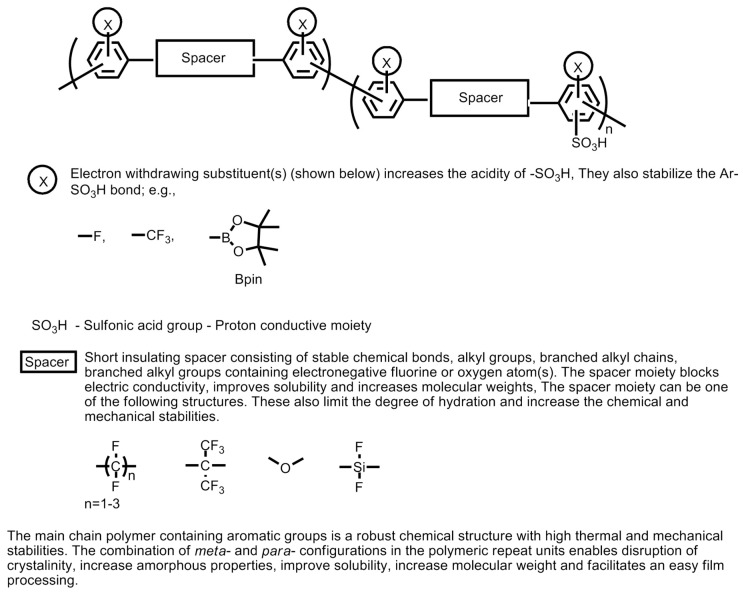
Strategy for developing novel sulfonic acid-containing polymers. Artwork adapted from the descriptions given in the Ref. [4].

On the other hand, the properties of sulfonated poly(aryl sulfones) can be further improved by (a) incorporating sulfonic acid groups into the pendant chain fluorination of the main polymeric chain; (b) substitution of the ether groups on the parent polymer chain by thioether group (e.g., poly(arylenethioethersulfone)-type sulfonated poly(aryl sulfone) polymers); (c) the incorporation of additional pendant groups; (d) incorporating additional co-polymeric moieties; and (e) incorporating inorganic fillers composites. The strategy for developing novel thermally and chemically stable sulfonic acid-containing polymers is explained and summarized in Figure 25 [4].

#### 6.4.6. Natural Polymer-Based Solid Polymer Electrolytes

**Figure 26:**
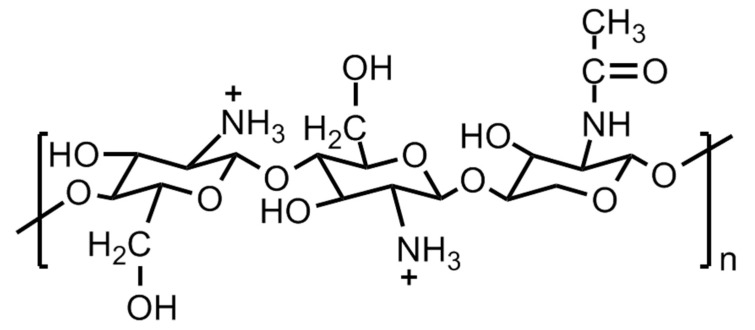
Molecular structure of chitosan.

Natural polymers, such as pectin, chitin phosphate, chitosan, gelatin, agar, alginic acid, plant cellulose, uracil, bacterial cellulose, etc., are inexpensive, abundantly available, environmentally friendly and potentially promising candidates for developing solid polymer electrolyte for fuel cell applications. In this direction, cellulose and its derivatives-based polymer membranes have been extensively explored by various research groups [126,127,128,129,130]. One such example is [2-acrylamido-2-methyl-1-propane sulfonic acid (AMPS)]-grafted bacterial cellulose membranes, which demonstrated proton conductivity values close to Nafion. Another natural polymer extensively investigated for a potential solid polymer electrolyte is chitosan. The structure of chitosan is shown in Figure 26. Chitosan, upon hydration, acts as a cationic polymer electrolyte. The conductivity in these polymers takes place by means of hydroxide (OH^−^) transport. The polymer backbone chain bears weak alkaline –NH_2_ groups which undergo partial protonation upon hydration (NH_2_ + H_2_O ↔ NH_3_ + OH^−^). Although the chitosan polymeric material is largely crystalline with clusters of amorphous areas randomly scattered, the latter are the sites where the OH^−^ transport takes place. The intrinsic conductivity of these polymer electrolytes is very low ~10^−4^ Scm^−1^ and considered to be inadequate for a fuel cell to function appropriately. The protonic conductivity of the chitosan-based polymer electrolytes can be increased by incorporating proton-donating groups, such as (a) heteropolyacids and acid salts, and (b) inorganic fillers, e.g., acidic oxide particles, ammonium salts, acidic groups. It has been observed that the additives enhance the proton conductivity of the material by compromising the mechanical stability. The latter can be improved by introducing crosslinking in the polymer chain led by covalent or ionic moieties, acidic groups, polybasic acids, etc. As the ion transport mainly takes place in the amorphous regions of the material, the amorphization of the chitosan is considered to be an advantage. The amorphization is achieved by using salts, plasticizers, covalent crosslinking by glutaraldehyde, oligo-D,L-lactic acid, and substituting large and bulky groups like the butyryl group in the places of OH^−^ groups on the parent chain. The chitosan-acid-mixed polymer membranes act like acid-based composites and demonstrate proton conduction upon hydration. These membranes exhibit conduction up to 160 °C at low hydration. However, chitosan-based polymer membranes show low proton conductivities, but, these demonstrate substantially restricted permeability towards methanol compared to Nafion [131,132]. The scientific reports on chitosan-based polymer materials for solid electrolyte applications have created huge possibilities for the other naturally occurring polymers to be investigated for suitable modification and application in solid polymer electrolytes for fuel cells.

#### 6.4.7. Polymers for Alkaline Fuel Cells

**Figure 27:**
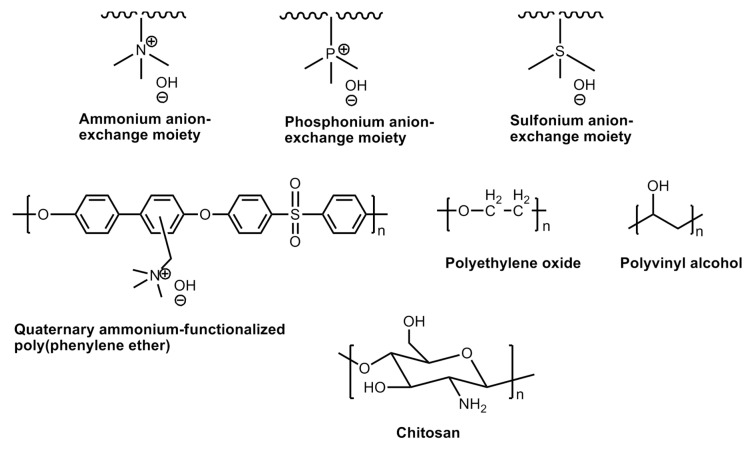
Molecular structures of anion exchange moieties and anion exchange polymers.

An alkaline fuel cell uses aqueous KOH as an electrolyte medium. Unlike cation fuel cells, it does not require the incorporation of noble metal catalysts. Thus, these types of fuel cell structures are completely noble metal-free. The main challenge of the alkaline fuel cell materials is the sensitivity of the liquid KOH towards CO_2_ which enter into the cell compartment either from fuel or the air. The latter causes the precipitation of crystalline K_2_CO_3_ which blocks the porous electrodes. When the alkaline anion exchange membranes are employed as electrolyte material, the mobile cations (e.g., K^+^) are not present at the vicinity, so the formation of crystal carbonates is eliminated. However, the possibility of carbonation is not ruled out which is believed to decrease the conductivity of the membrane. In recent years, a number of polymers have been developed and the molecular structures of some noteworthy polymers are shown in Figure 27. Quaternary ammonium-functionalized polymers are popular in this regard. These were discovered during early 1960s and have been widely used in various electro-dialysis processes. The membranes were observed to be demonstrating good anion conductivities; the stabilities were not satisfactory owing to the nucleophilicity of the hydroxide ions. On the other hand, polysulfone-based polymers have shown greater stabilities. Alternately, the incorporation of aqueous hydroxide salts into the neutral polymer matrix gives rise to more stable polymeric material conducting hydroxide ions. These polymers possess Brønsted base functionalities, *viz*., oxygen, nitrogen or sulfur. These interact with the cationic part of the hydroxide salts. Some of the popular polymers in this category are polyethylene oxide (PEO), chitosan, and polyvinyl alcohol (PVA) [133,134,135].

The protonic conductivity ranges of the polymer electrolyte membranes along with their relative merits are summarized in the table below (Table 1).

## 7. Mechanism of Proton Transfer in Proton Conducting Solid Polymer Electrolytes

**Figure 28:**
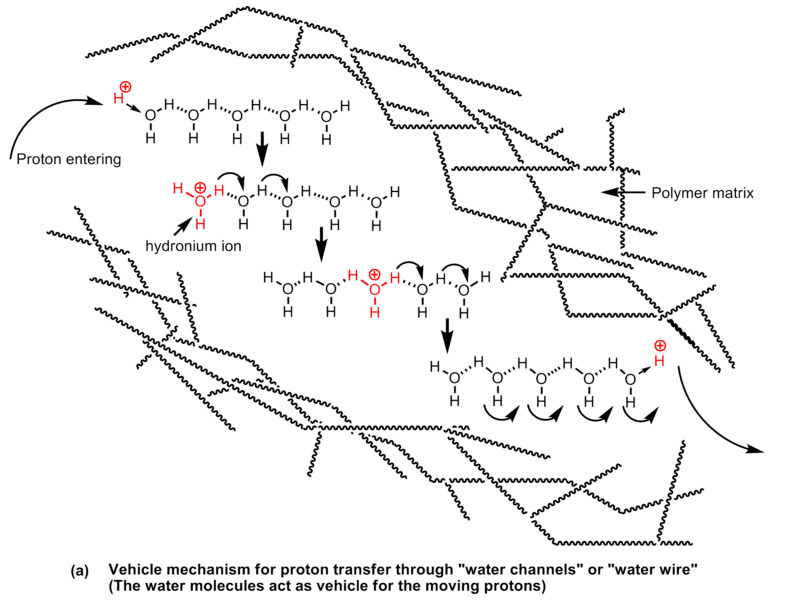

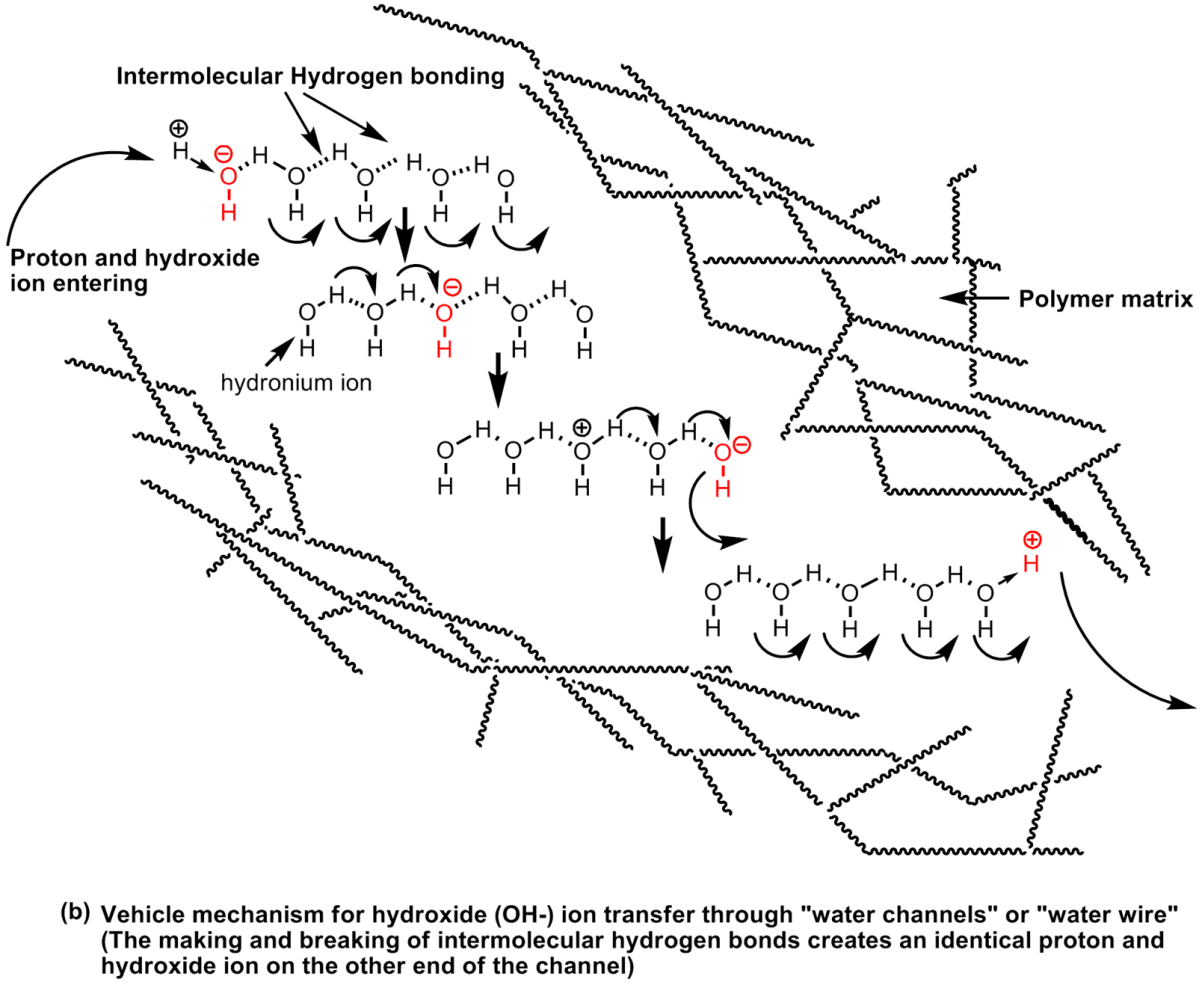
Vehicle mechanism of (**a**) proton, and (**b**) hydroxyl ion transport through water channels.

It is apparent that proton conductivity is the fundamental aspect of polymer electrolyte membranes, which that a solid electrolyte and the conduction of protons is largely regulated by (a) the presence of hydrophilic groups on the polymer chain, and (b) the hydration of the polymers (i.e., presence of water molecules). The proton mobility in water is exceptionally high. The conductance is seven times higher than Na^+^ and ~five times higher than K^+^ at room temperature [136]. A water molecule has a tetrahedral geometry whereas an oxygen atom sits in the center and is bonded with two hydrogen atoms at the alternate corners of a cubic structure and the other two alternate corners are occupied by oxygen’s lone pairs of electrons. The oxygen atom is electronegative and creates partial positive charges on the hydrogens bonded to it. This creates the possibility of the formation of inter-, as well as intra-molecular hydrogen bonds between the electronegative atoms on the hydrophilic groups present in the polymeric material, or other water molecules in the vicinity as shown in Figure 28.

An addition of a proton at the end of such a chain of water molecules forming a network via intermolecular hydrogen bonds forming a configuration resembles a conductive wire or “water wire” or “water channels” or “water string”. The proton transport in the proton conducting polymer-electrolytes takes place via two main transport mechanisms, *viz*., (a) the vehicle mechanism, and (b) proton-hopping or proton-jumping mechanism, also known as Grotthuss mechanism. The vehicular mechanism is believed to be relevant in high humidity and low temperature operational conditions. In this mechanism, the water molecules come together to form clusters closer to the electrolyte membrane surface and the formation of proton transporting water channels or “water-wires” takes place. The water molecules act as a vehicle for transporting protons from one end of the channel to the other. In Nafion type polymer membranes, the proton conduction mainly takes place by vehicle mechanism. Nafion is poly(tetrafluoroethylene) sulfonic acid-based polymers in which the hydrophilic acid groups (sulfonic acid functional groups) are arranged at certain intervals across the polymer backbone. The hydrophobic fluorocarbon domains and the hydrophilic sulfonic acid domains on the polymer chain possess widely different polarities resulting in phase separation in the range of the nanoscale dimension. The formation of water clusters at hydrophilic domains and in the subsequent stages, the formation of nanostructured-channels and the network of channels carries out proton transport. At high temperatures, the humidity begins to decline owing to the formation of vapors and steam and the channels are broken down or disrupted [54].

**Figure 29:**
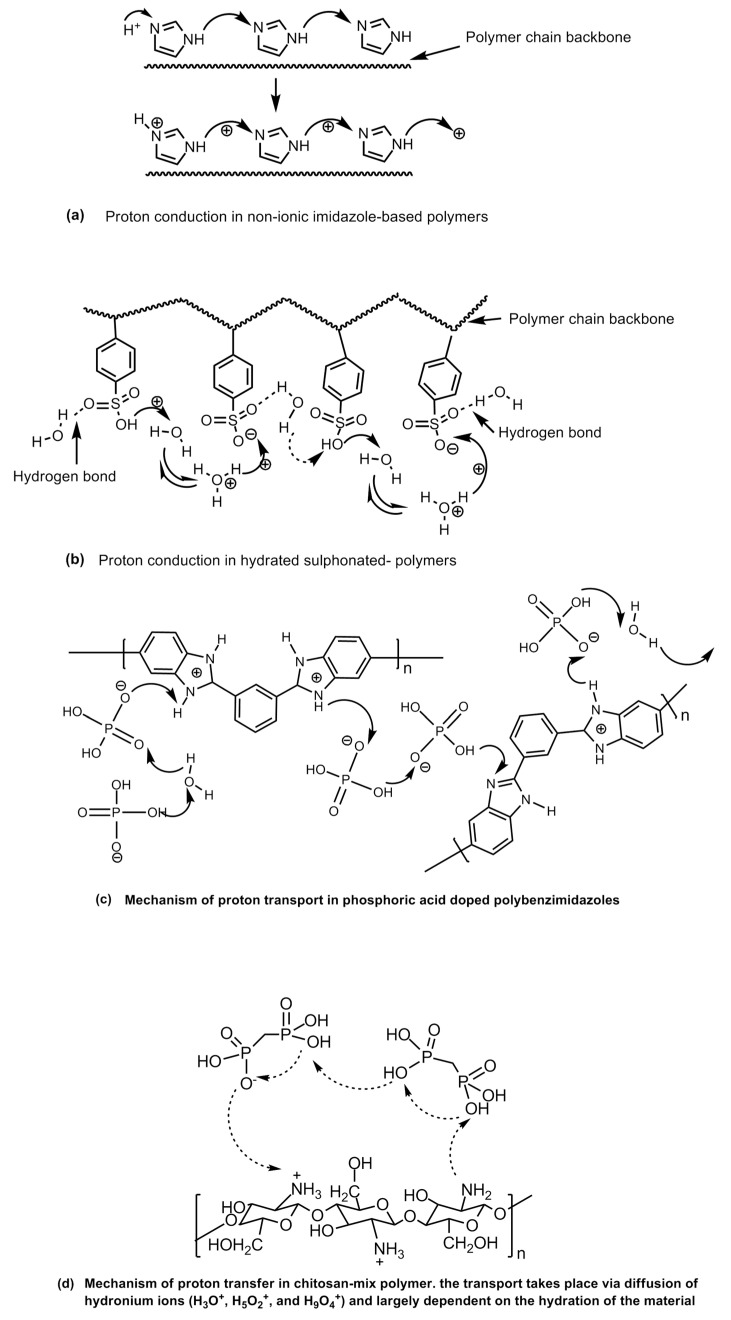
Proton-hopping or Grotthuss-type mechanism in proton conducting solid electrolyte polymers; (**a**) Proton conduction in non-ionic imidazole-based polymers, (**b**) hydrated sulphonated- polymers, (**c**) phosphoric acid doped polybenzimidazoles, and (**d**) chitosan-mix polymer, where the transport takes place via diffusion of hydronium ions.

When the water clusters are no longer connected, the proton conduction stops. In the proton hopping or Grotthuss type mechanism, the protons are transported via charge carrying functional groups (the hydrophilic sulfonic acid functional groups) present on the polymer through the making and breaking of hydrogen bonds in a complex process (Figure 28). At low humidity and high temperatures, when the vehicle mechanism fails, this transport mechanism explains the proton conductivity by the polymer electrolyte membrane satisfactorily. The sulfonated-polymer-based membranes as well as the polymers containing nitrogen heterocycles and hybrid polymer composite-based membranes which demonstrate appreciable proton conducting at low humidity levels and high temperatures are explained to transport protons via a proton-hopping mechanism. The proton-hopping or Grotthuss-type mechanism in different types of proton conducting solid electrolyte polymers have been shown in Figure 29.

In the past four to five decades, the hydrogen fuel cell demonstration units have been working with sulfonated perfluoro polymers, e.g., Nafion. These polymers offer satisfactory proton conducting properties but possess several drawbacks, such as (a) low proton conductivities at low hydration and high temperatures (>373 K), (b) high hydration levels showing the swelling of the polymer resulting into low mechanical stability, (c) high cost and (d) inadequate mechanical and chemical stabilities. Several polymeric materials have been developed since the discovery of Nafion membranes along with the improvement in the functional properties at considerably reduced costs, e.g., the incorporation of hydrophilic and hydrophobic functional groups in varying amounts and fine tuning their positions on the polymer backbone. The hydrophilic groups are responsible for proton conduction whereas the hydrophobic polymer backbone contributes in chemical and mechanical stability. The anion exchange polymer electrolyte membranes for alkaline fuel cells are still in their early phase of development and the main challenge of these materials is durability along with cost economy. The research on proton conducting solid electrolyte polymer membranes has entered in its advanced stage of research, where the materials are now tested for long operational hours ranging between 10,000 and 60,000 h, e.g., perfluorosulfonic acid-based polymers.

## 8. Conclusions

We discussed the conducting polymers in the application of hydrogen storage and fuel cells. Polyaniline has been explored the most for hydrogen storage experiments. The synthesis processes for the conducting polymers like polyaniline, polypyrrole and polythiophene can be fine-tuned to obtain the desired adsorbent materials with appropriate morphological and functional properties. This review also summarizes the development and current status of polymer-based solid electrolyte membranes. Nafion (PFSA) membranes have been reported for its outstanding performance and displayed an edge over other proton conducting solid electrolyte membranes due to its entire set of balanced properties. However, its manufacturing cost is quite high, and leaves plenty of opportunities as well as possibilities for the development of newer and novel materials for commercial purposes. The future of proton as well as anion conducting solid electrolyte polymer membranes for the next generation hydrogen fuel cells is optimistic and capable of bringing the humankind workable solutions for achieving pollution free energy materials and technologies.

## Figures and Tables

**Table 1:** Summary of proton conducting solid electrolyte membranes for hydrogen fuel cells.

Proton Conducting Polymers	Conductivity Range (Scm^−1^)	Relative Merits
**Polyperflurosulfonic acid (PFSA) Nafion-type membranes**		
Nafion (Dupont); Aciplex and Flemion (Asahi Chemical Company); AQUIVION (Solvay Solexis); Flemion (3 M^TM^ Asahi Glass); Aciplex (Asahi Kasei); fumionF (FuMA-Tech)	0.1 at RT	Stable in redox environment; excellent mechanical strength; high proton conductivity; durable (up to 60,000 h); high operating cost; continuous hydration is required
**Partially fluorinated polystyrene-based membranes**		
Sulphonated α,β,β-trifluorostyrene; m-trifluoromethyl α,β,β-trifluorostyrene; Sulphonated polymer of α,β,β-trifluorostyrene; Sulphonated copolymer of α,β,β-trifluorostyrene; Copolymer of α,β,β-trifluorostyrene BAM membranes	5 × 10^−2^ to 9 × 10^−2^	Stable in oxidative and reductive environment; excellent mechanical strength; rapid material degradation; comparable proton conductivity to PFSA; durable but less than PFSA; slightly less operating cost as PFSA but still too high; hydration requirement can be tuned based on the mixing/adding of external molecule; slimmer membrane with less interlinked water-filled network; less sulfonic acid groups
**Polystyrene-based membranes**		
PVDF (polyvinylidine fluoride) –*grafted*–PSSA; Teflon–*grafted*–PSSA; (low density polyethylene-*grafted*–PSSA, [tetra fluoroethylene–*co*–perfluoroporpylene (FEP, Teflon 100)] –*grafted*–PSSA; PSSA-(*co*–polymer) –polymers	0.001 to 0.24	Less stable in oxidative and reductive environment; reduced ion; low cost membrane; reduced ion exchange capacity and conductivity at high rates; low cost membrane; slimmer membrane with less interlinked water-filled network; less sulfonic acid groups
**Sulfonated polyimide (SPI)-based membranes**		
Linear polyimide; aromatic heterocyclic polyimide; phthalic-and naphthalenic-type sulfonated polyimides Sulfonated polyimide-graphene composites	1.67 (at 393.15 K); 1.20 (at 353.15 K)	High mechanical strength; highly thermostable; chemically stable; increased proton conductivity; good durability; instability in hydrated state which can be improved by mixing imide group
**Polybenzimidazole (PBI)-based membranes**		
Phenylene-based PBI, poly(2,5 benzimidazole), and pyridine-based PBI polymers; (PBI/H3PO4) [Commercial polymers: CeltecL, CeltecP1000, and CeltecV from BASF]; Poly-[2,2′-(m-phenylene)-5,5′-bibenzimidazole] (PBI); poly(2,5-benzimidazole) (ABPBI)	10^−5^ to 0.15 at 433.15–453.15 K	High thermal stability; good chemical resistance; high oxidative and thermal stability; presence of a liquid electrolyte; good proton conductivity; durable; stable in hydrated state; unsuitable for portable devices
**Polyphosphazene-based Membranes**		
Sulfonated [bis(3-methyl phenoxy)] phosphazanes; phosphonic functionalized Ppz (P-Ppz);polybenzimidazole-Polyphosphazenes	1 × 10^−2^ to 12 × 10^−2^	Polymer properties in terms of its mechanical stability; the latter can be improved by introducing crosslinking with other polymers; low permeability to methanol; polymer chain possesses high tortional mobility
**Sulfonated aromatic hydrocarbon polymer -based membranes**		
Sulfonated poly(arylene ether ketone)s or SPAEKs; sulfonated poly(ether ether ketone)s or SPEEKs; sulfonated poly-sulfones; sulfonated polyethersulfones; sulfonated polyphenylsulfones; sulfobutylated polyethersulfonates	0.01 to 0.048	Good mechanical strength; chemically and thermally stable even at elevated temperature; high proton conductivity which can be tuned with polar/sulfonic acid groups; durability still to be tested
**Natural polymer-based solid polymer electrolytes**		
[2-acrylamido-2-methyl-1-propane sulfonic acid (AMPS)]-grafted bacterial cellulose membranes; sulfonated chitosan-based polymer	<10^−4^	Mechanically stable; low conductivity but can be increased by incorporating proton donating groups which lowers the mechanical strength.
**Polymers for alkaline fuel cells**		
Quaternary ammonium-functionalized polymers; polyethylene oxide (PEO); chitosan; polyvinyl alcohol (PVA); fluorinated poly(tetrafluoroethylene-*co*-hexafluoropropylene) (FEP); and the alkaline partially fluorinated poly(ethylene-*co*-tetrafluoroethylene) (ETFE) membranes	0.012 to 0.078	Less stability; high degradation rates; good anion conductivities; neutral polymer matrix with aqueous hydroxide salts form more stable polymeric materials
